# Introgression from Domestic Goat Generated Variation at the Major Histocompatibility Complex of Alpine Ibex

**DOI:** 10.1371/journal.pgen.1004438

**Published:** 2014-06-19

**Authors:** Christine Grossen, Lukas Keller, Iris Biebach, The International Goat Genome Consortium, Daniel Croll

**Affiliations:** 1 Institute of Evolutionary Biology and Environmental Studies, University of Zürich, Zürich, Switzerland; 2 Department of Zoology, University of British Columbia, Vancouver, Canada; 3 Kunming Institute of Zoology, Chinese Academy of Sciences, State Key Laboratory of Genetic Resources and Evolution, Kunming, China; 4 INRA, UMR444, Laboratoire de Génétique Cellulaire, Castanet-Tolosan, France; 5 Michael Smith Laboratories, University of British Columbia, Vancouver, Canada; Aarhus University, Denmark

## Abstract

The major histocompatibility complex (MHC) is a crucial component of the vertebrate immune system and shows extremely high levels of genetic polymorphism. The extraordinary genetic variation is thought to be ancient polymorphisms maintained by balancing selection. However, introgression from related species was recently proposed as an additional mechanism. Here we provide evidence for introgression at the MHC in Alpine ibex (*Capra ibex ibex*). At a usually very polymorphic MHC exon involved in pathogen recognition (*DRB* exon 2), Alpine ibex carried only two alleles. We found that one of these *DRB* alleles is identical to a *DRB* allele of domestic goats (*Capra aegagrus hircus*). We sequenced 2489 bp of the coding and non-coding regions of the *DRB* gene and found that Alpine ibex homozygous for the goat-type *DRB* exon 2 allele showed nearly identical sequences (99.8%) to a breed of domestic goats. Using Sanger and RAD sequencing, microsatellite and SNP chip data, we show that the chromosomal region containing the goat-type *DRB* allele has a signature of recent introgression in Alpine ibex. A region of approximately 750 kb including the *DRB* locus showed high rates of heterozygosity in individuals carrying one copy of the goat-type *DRB* allele. These individuals shared SNP alleles both with domestic goats and other Alpine ibex. In a survey of four Alpine ibex populations, we found that the region surrounding the *DRB* allele shows strong linkage disequilibria, strong sequence clustering and low diversity among haplotypes carrying the goat-type allele. Introgression at the MHC is likely adaptive and introgression critically increased MHC *DRB* diversity in the genetically impoverished Alpine ibex. Our finding contradicts the long-standing view that genetic variability at the MHC is solely a consequence of ancient trans-species polymorphism. Introgression is likely an underappreciated source of genetic diversity at the MHC and other loci under balancing selection.

## Author Summary

The major histocompatibility complex (MHC), a crucial component of the defense against pathogens, contains the most polymorphic functional genes in vertebrate genomes. The extraordinary genetic variation is generally considered to be ancient. We investigated whether a previously neglected mechanism, introgression from related species, provides an additional source of MHC variation. We show that introgression from domestic goat dramatically increased genetic variation at the MHC of Alpine ibex, a species that had nearly gone extinct during the 18^th^ century, but has been restored to large numbers since. We show that Alpine ibex share one of only two alleles at a generally highly polymorphic MHC locus with domestic goats and that the chromosomal region containing the goat-type allele has a signature of recent introgression. Our finding contradicts the long-standing view that ancient trans-species polymorphism is the sole source of the extraordinary genetic variability at the MHC. Instead, we show that in Alpine ibex introgression generated genetic diversity at a MHC locus. Our study supports the view that loci favoring genetic polymorphism may be susceptible to adaptive introgression from related species and will encourage future research to identify unexpected signatures of introgression.

## Introduction

The MHC is one of the most gene-dense regions and contains the most polymorphic functional genes in vertebrate genomes [1]–[3]. The major role of MHC gene products is the recognition of foreign peptides and their presentation to specialist immune cells in order to initiate an immune response [4]. Specific MHC haplotypes and MHC heterozygosity were shown to be associated with immunity to diseases [e.g. 5–9]. A higher allelic diversity at MHC loci is expected to be favored because individuals with a broader range of MHC sequences (binding and presenting a broader range of pathogenic peptides) should be able to more successfully fight diseases. However, the mechanisms generating and maintaining the extraordinary MHC diversity are not fully understood [reviewed in 10–12]. Three types of balancing selection, mediated by pathogen-driven or sexual selection, are usually invoked to explain MHC polymorphism: heterozygote advantage, negative frequency-dependent selection and fluctuating selection [11], [13]. Linked recessive deleterious mutations may additionally contribute to a pattern of balancing selection and may explain why MHC alleles are frequently more highly diverged from each other than expected otherwise [12]. While MHC alleles often exhibit high sequence divergence, balancing selection tends to even out allele frequencies among populations and hence such loci show lower population differentiation than neutral loci [14].

Balancing selection at a locus may predate speciation events and maintain a set of highly divergent alleles termed ancient trans-species polymorphism [15]. Adaptive genetic variation at loci under balancing selection is generally assumed to stem from standing genetic variation or mutations. However, adaptive genetic variation may also be generated through introgression, the gene flow between species [reviewed in 16]. Introgression at loci under balancing selection is expected to be favored because of the selective advantage of rare alleles [14], [16]. Therefore, loci under balancing selection are good candidates for adaptive introgression as shown for self-incompatibility genes in plants [17] and a coat pattern gene in animals [18].

Recently, introgression was proposed as an additional mechanism contributing to high levels of genetic diversity at the MHC [19]. Due to the significance of MHC variation for the defense against infectious diseases, introgression at the MHC is likely adaptive. Introgression from archaic humans was suggested to have shaped the human MHC [20]. MHC diversity in domestic mammals may have been augmented by introgression from wild ancestors [21]. Several MHC alleles were found to be shared between two newt species consistent with introgression among species [22]. However, direct evidence for recent introgression events at the MHC in wild species is lacking. Here we present evidence that introgression from domestic goat was an important source of MHC class II variation in Alpine ibex.

The Alpine ibex (*Capra ibex ibex*) is a species of wild goat occupying high-alpine niches of the European Alps spanning from Northern Italy and France to Slovenia. Several related ibex species are found in mountain ranges of Southern Europe, Central Asia, Northeast Africa, and the Arabian Peninsula (the Mountain goat of North America, *Oreamnos americanus*, belongs to a different genus). One of these species, the bezoar (*Capra aegagrus*) is the ancestor of the domestic goat [23]. Following near extinction during the 18^th^ century due to overhunting, Alpine ibex were reintroduced to most parts of the European Alps from the only remaining population in Northern Italy (Gran Paradiso National Park). The reintroduction was very successful, and the species has recovered to more than 40'000 individuals living across the European Alps. Therefore, the Alpine ibex is considered a flagship species of the restoration of large mammals. However, the re-introduction caused several bottlenecks of less than 100 individuals, which substantially depleted genetic variability [24]–[26]. The depletion of genetic variability is particularly striking at the *DRB* locus of the MHC class II. Only two alleles (Caib-DRB*1 and Caib-DRB*2) were reported at the exon 2 of *DRB* in at total of 125 individuals [27], [28]. In comparison, both the domestic goat (*C. aegagrus hircus*) and its wild ancestor the bezoar (*C. aegagrus*) are highly polymorphic at this exon. We show that introgression from domestic goat is responsible for the fact that Alpine ibex are polymorphic at all at the exon 2 of the *DRB* locus, suggesting that introgression can be an important evolutionary force shaping the evolution of the MHC.

## Results and Discussion

**Figure 1:**
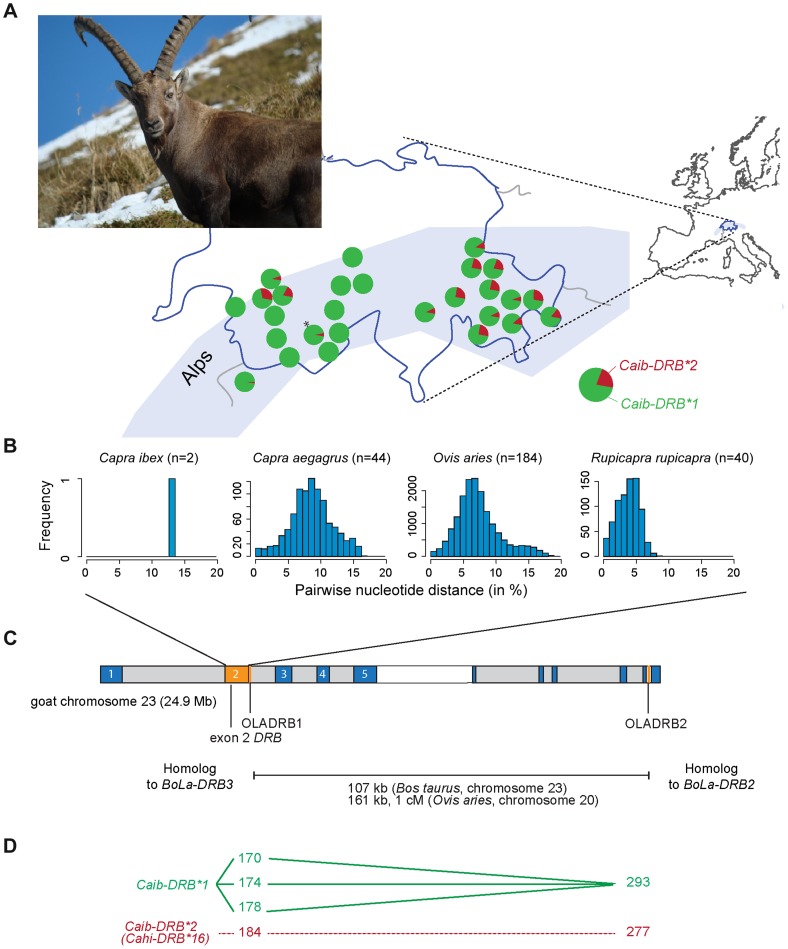
MHC *DRB* allele diversity in Alpine ibex and chromosomal localization of genetic markers. (A) Frequency distribution of the two MHC *DRB* exon 2 alleles in Alpine ibex populations across the Swiss Alps. The marker OLADRB1 was used to assess the frequency of Caib-DRB*2. All populations with n≥12 are shown except for Weisshorn (n = 9; marked by an asterisk). See Table S1 for complete allele frequency data. (B) Genetic diversity of MHC *DRB* alleles in different Caprinae species. The proportions of sites that differ between each pair of sequences are shown based on 227 bp sequence length. (C) Chromosomal locations of MHC genes and genetic markers were mapped to the goat chromosome 23 using the homologous cattle (*Bos taurus*) chromosome 23 as a reference (for further details see [40]). The MHC *DRB* exon 2 is shown in orange. Marker distances between the microsatellites OLADRB1 and OLADRB2 are based on cattle chromosome 23 and sheep chromosome 20. (D) Observed linkage disequilibrium (connected lines) between sequence and microsatellite alleles. Caib-DRB*2 was completely associated to allele 184 of microsatellite OLADRB1 (previously shown for a subset of 98 individuals in Alasaad et al. [28]) and allele 277 of OLADRB2 (except 2 individuals out of 707).

We extended the sequencing by Schaschl et al. [27] and Alasaad et al. [28] from 125 to 203 Alpine ibex from different populations and did not find additional alleles at the exon 2 of the MHC *DRB* locus. We found that the second allele (Caib-DRB*2) was identical to the Cahi-DRB*16 allele of the domestic goat first reported from the Japanese breed Shiba ([29], Genbank accession AB008361). We will refer to *Caib-DRB* exon 2 sequence variants (236 bp) as *Caib-DRB* alleles throughout the manuscript (see Figure 1). We found no recombinant between the two alleles in Alpine ibex, although recombinants at this locus were found in several related ungulate species [27], [30], [31]. This suggests that the two alleles are not both ancient alleles of the Alpine ibex *DRB* locus.

We investigated the geographic distribution of the *Caib-DRB* alleles by genotyping the microsatellite marker OLADRB1 (Figure 1A). OLADRB1 is directly adjacent to the exon 2 of *DRB* in even-toed ungulates [32] and is in complete linkage disequilibrium (i.e. diagnostic) in Alpine ibex [28 and this study]. We found substantial variation in *Caib-DRB* allele frequencies among 40 Alpine ibex populations (n = 754 individuals). The frequency of the allele Caib-DRB*2 ranged from 0% (most populations of Central and South-Western Swiss Alps) to 31% (North-Western Swiss Alps; Figure 1A and Table S1). Caib-DRB*2 was generally at a high frequency in the Eastern Swiss Alps (Figure 1A). In the founder population for the reintroductions, the Gran Paradiso National Park in the Italian Alps, the frequency of Caib-DRB*2 was 3%. The striking population structure observed at *Caib-DRB* reflects the complex reintroduction history shown by neutral markers [25]. Individuals were first brought from the Gran Paradiso to Swiss zoos. From these zoos, initial reintroductions established three wild populations across the Swiss Alps, which in turn served as source populations for subsequent reintroductions across Switzerland and elsewhere.

### The origin of the Caib-DRB*2 allele in Alpine ibex

The MHC is known to harbor genetically divergent alleles within species [4], [33]. The two *Caib-DRB* alleles found in Alpine ibex were even more divergent than expected from related species (13.2% nucleotide and 22.6% amino acid difference, Figure 1B). In comparison, the mean pairwise nucleotide difference among MHC *DRB* exon 2 alleles found in domestic goats and their wild ancestors (bezoar) was substantially lower (8.6%, 95% confidence interval CI 1.8–15.0%; 17.8% amino acid difference, Figure 1B). Domestic sheep (*Ovis aries*) and Chamois (*Rupicapra rupicapra*) showed even lower mean allele divergence (Domestic sheep: nucleotide divergence 7.4%; 95% CI: 2.2–15.9% and amino acid divergence 15.0%; Chamois: nucleotide divergence 3.9%; 95% CI: 0.9–6.6% and amino acid divergence 9.5%; Figure 1B, Table S2). The high genetic divergence between the two Alpine ibex *Caib-DRB* alleles and the identity of Caib-DRB*2 to an allele identified in domestic goats may be indicative of introgression from domestic goats into Alpine ibex. The two species share a common ancestor 2–6 million years ago [34], [35] and hybrids, which can survive and breed have repeatedly been reported in the wild [36]. Alternatively, related species may show similarities at MHC alleles because of ancient trans-species polymorphisms caused by balancing selection [11], [15], [37], [38].

We assessed the likelihood that two species share identical MHC *DRB* exon 2 sequences by analyzing 112 sequences of length identical to the two Alpine ibex MHC *DRB* exon 2 sequences (236 bp) from eight species of the Caprinae subfamily comprising domestic goats and Alpine ibex. With the exception of the sequence shared between domestic goats and Alpine ibex, we found no shared alleles among species. However, we found evidence of shared sequences among species by including sequences of shorter minimum length (227 bp; n = 332) representing 11 species. Six pairs of species shared 13 alleles (see Table S3). For all except one of these species pairs, hybridization has either been observed or signatures of past hybridization between the species have been reported (see Table S3 for more details).

Hybrids between Alpine ibex and domestic goats were occasionally reported in the past and microsatellite analyses confirmed the existence of F1 hybrids (see Figures S1, S2 for more details). However, in a survey of Alpine ibex individuals, we found no evidence of recent hybrids based on genetic clustering analyses using the software STRUCTURE [39]. For this, we analyzed 30 neutral microsatellites in 1781 individuals [this study and 25] and 546 SNPs in 95 individuals. Thus, the presence of Caib-DRB*2 in Alpine ibex in extant populations is not solely due to recent hybrids.

### Alpine ibex share nearly identical *DRB* intron and exon sequences with a domestic goat breed

**Figure 2:**
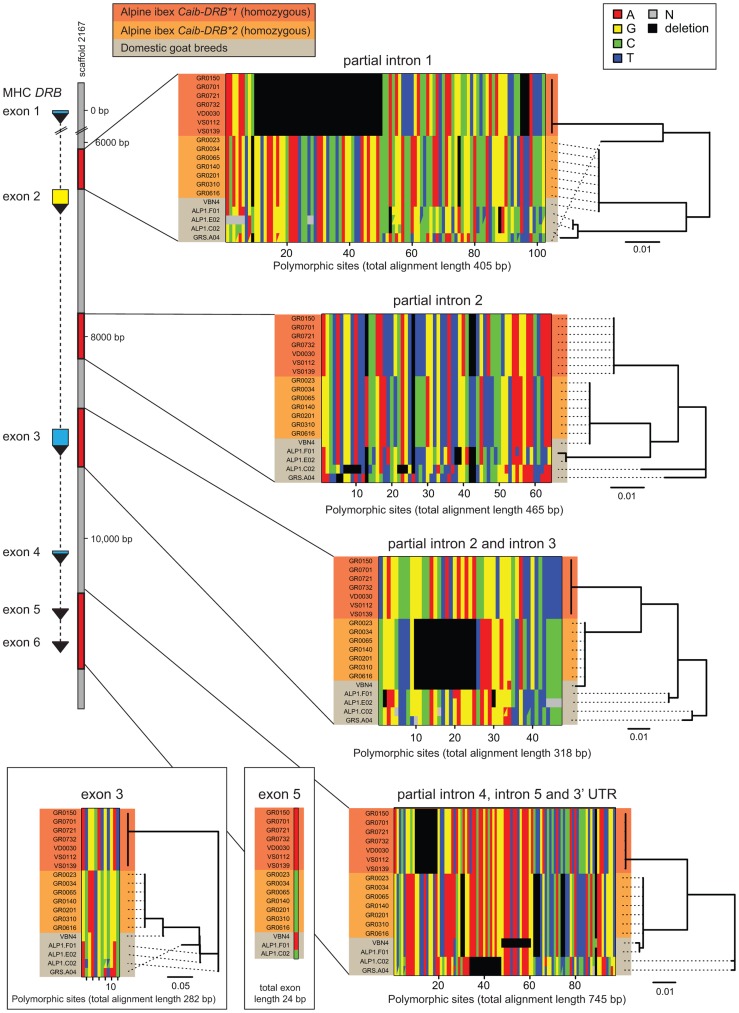
Sequence alignments of coding and non-coding regions of the MHC *DRB* gene in Alpine ibex and domestic goat. The *DRB* gene (11 kb in domestic sheep) is located on to the goat reference genome scaffold2167 at position 79'094 bp. A total of 2253 bp of the MHC *DRB* gene was sequenced including partial sequences of introns 1–4 and the complete intron 5 as well as a 3′ UTR sequence and complete sequences of exons 3, 5 and 6. Exon 6 is not shown as no polymorphism was found. Seven Alpine ibex homozygous for Caib-DRB*1 (red), seven Alpine ibex homozygous for Caib-DRB*2 (orange) and five domestic goat individuals selected from a screening of diverse breeds (grey) were sequenced. All seven Alpine ibex homozygous for Caib-DRB*2 were homozygous for the same haplotype at all four loci. This haplotype was highly distinct from the haplotype carried by all seven Alpine ibex homozygous for Caib-DRB*1. The domestic goat VBN.4 shared a nearly identical haplotype with individuals homozygous for Caib-DRB*2 at all four sequenced loci (99.8% sequence identity across 2253 bp). The domestic goat VBN.4 was homozygous for the *DRB* exon 2 Cahi-DRB*16, which is identical to Caib-DRB*2 (not shown). The phylogenetic trees are based on neighbor-joining. See Supporting Text S1-S4 for full sequence alignments.

Introgression is expected to generate highly similar sequence tracts between the donor and recipient species [16]. If introgression was the source of Caib-DRB*2, we predict that in addition to the *DRB* exon 2, intronic and other non-coding regions should be highly similar between Alpine ibex carriers of Caib-DRB*2 and domestic goats. In contrast, if ancient trans-species polymorphism was responsible for maintaining identical *DRB* exon 2 sequences, we predict that the surrounding non-coding regions should have accumulated significant sequence divergence between Alpine ibex and domestic goats. In order to distinguish between these scenarios, we sequenced four regions of the *DRB* gene. The MHC region is located on chromosome 23 in domestic goats (Figure 1C) and the 11 kb *DRB* gene is fully contained on the 136 kb goat reference genome scaffold2167 [40]. The four sequenced regions comprise a total of 2253 bp and covered the complete sequences of exons 3, 5 and 6, the complete intron 5 as well as partial sequences of introns 1–4 and a 3′ UTR sequence (Figure 2, see Supporting Text S1–S4 for full sequence alignments). We sequenced seven Alpine ibex homozygous for Caib-DRB*1, seven Alpine ibex homozygous for Caib-DRB*2 and five domestic goat individuals selected from a screening of diverse breeds. All seven Alpine ibex homozygous for Caib-DRB*1 were homozygous for the same haplotype at all four loci and were strongly differentiated from Alpine ibex homozygous for the Caib-DRB*2 allele (5.1% nucleotide divergence). The haplotype of Caib-DRB*1 Alpine ibex individuals did not show any close similarity to sequences found in domestic goats (Figure 2).

All seven Alpine ibex homozygous for Caib-DRB*2 were homozygous for the same highly distinct haplotype (Figure 2). We found that the domestic goat individual VBN.4 of the breed Valais Blackneck carried a very similar haplotype (99.8% sequence identity across 2253 bp). The haplotypes of VBN.4 and of Alpine ibex homozygous for Caib-DRB*2 differed only by five SNPs at exon 3, intron 3, exon 5, and the region comprising partial introns 4, 5 and 3′ UTR sequences (Figure 2). The domestic goat VBN.4 was homozygous for the *DRB* exon 2 Cahi-DRB*16, which is identical to Caib-DRB*2. The high similarity in both coding and non-coding regions of the *DRB* gene between the domestic goat VBN.4 and Alpine ibex homozygous for Caib-DRB*2 strongly suggests domestic goat breeds were the donors of *DRB* sequences that introgressed into Alpine ibex.

We tested for the presence of recombinant sequences at the MHC *DRB*. For this, we concatenated sequences of all four loci and used the recombination test based on the Φw-statistic [41]. The five domestic goat sequences showed significant evidence for recombination (64 informative sites; *p*<0.0001). In contrast, the two haplotypes associated to the Caib-DRB*1 and Caib-DRB*2 allele, respectively, did not show any evidence for recombination (116 informative sites; *p* = 1). This suggests that Alpine ibex haplotypes associated with Caib-DRB*1 and Caib-DRB*2 alleles, respectively, did not co-exist for a long period in the populations.

### High expected heterozygosity extends to the chromosomal region surrounding *DRB*

**Figure 3:**
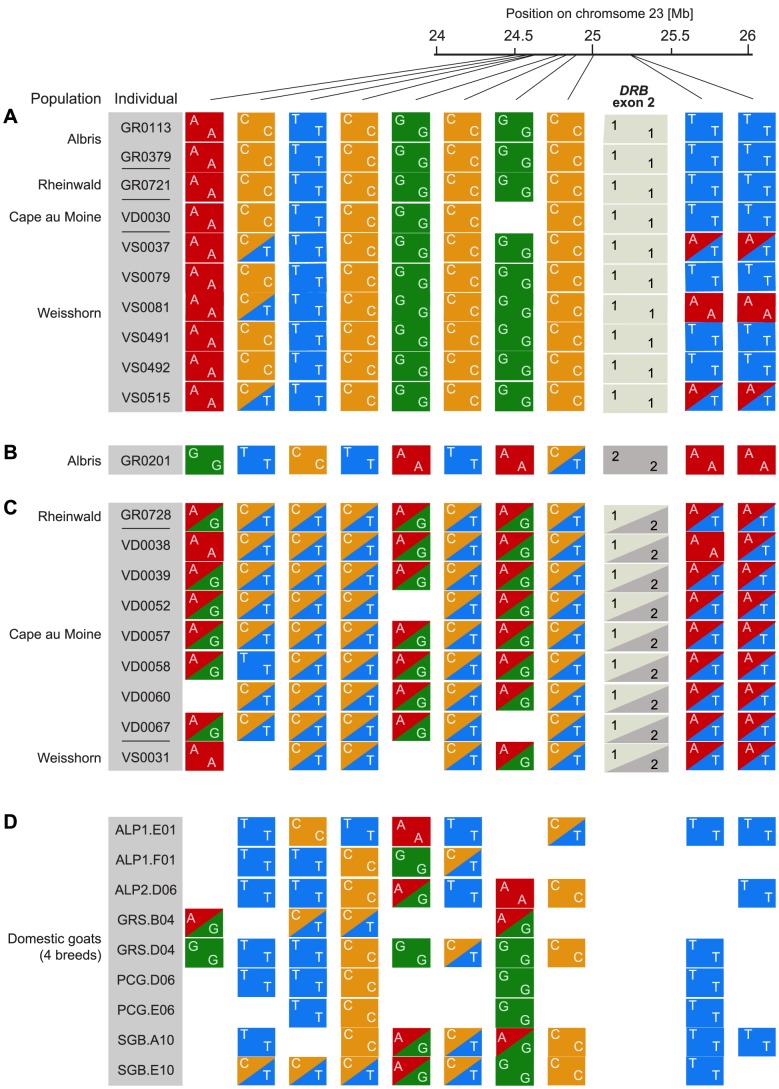
Alpine ibex and goat genotypes in 750*DRB*. RAD sequencing SNP genotypes in a 750(see Figure S3). A representative set of Alpine ibex individuals (A) homozygous for Caib-DRB*1 (n = 10) and (B and C) carrying Caib-DRB*2 (n = 10) are shown in the top panels. (D) The corresponding RAD sequencing SNP genotypes for 9 domestic goat individuals. SNPs with a minor allele frequency of less than 0.1 within Alpine ibex were excluded. Alpine ibex carrying Caib-DRB*2 are nearly exclusively heterozygous at the RAD sequencing SNP loci and share alleles both with Alpine ibex homozygous for Caib-DRB*1 and with domestic goats.

A recent introgression event from domestic goats into Alpine is expected to lead to a chromosomal region of high expected heterozygosity in individuals carrying the introgressed allele. To characterize the genomic region surrounding the *DRB* gene, we performed restriction site associated DNA sequencing (RAD-seq). We genotyped 15 Alpine ibex homozygous for Caib-DRB*1 and 15 Alpine ibex carrying Caib-DRB*2 of which 14 were heterozygous for Caib-DRB*2. The sampling covered individuals from four populations. Additionally, we included nine domestic goat individuals (representing four breeds). The RAD sequences were mapped to the domestic goat genome [40]. We identified 258 polymorphic SNPs located between 22 Mb and 27 Mb on chromosome 23. Eighty-six of these SNPs were polymorphic among Alpine ibex. Individuals carrying Caib-DRB*2 showed high rates of expected heterozygosity in a region of about 750 kb surrounding the second exon of *DRB* (24.5 to 25.25 Mb) on chromosome 23 (Figure S3). Furthermore, we found high sequence similarities to domestic goat sequences in the same region (for a representative sample of genotypes and SNPs see Figure 3). At 7 out of 10 SNPs, we found an allele that was shared between domestic goats (Figure 3D) and Alpine ibex carrying Caib-DRB*2 (Figure 3C) but not with Alpine ibex homozygous for Caib-DRB*1 (Figure 3A). Similarly, the Alpine ibex GR0201 homozygous for Caib-DRB*2 was homozygous for a SNP allele that was only found in domestic goats and other Alpine ibex carrying Caib-DRB*2 at 6 out of 10 SNPs (Figure 3B). These observations are consistent with introgression at the MHC *DRB* locus and indicate that introgression has increased genetic diversity of Alpine ibex in this genomic region.

### Alpine ibex haplotypes carrying Caib-DRB*2 are highly similar in the region surrounding the MHC region

**Figure 4:**
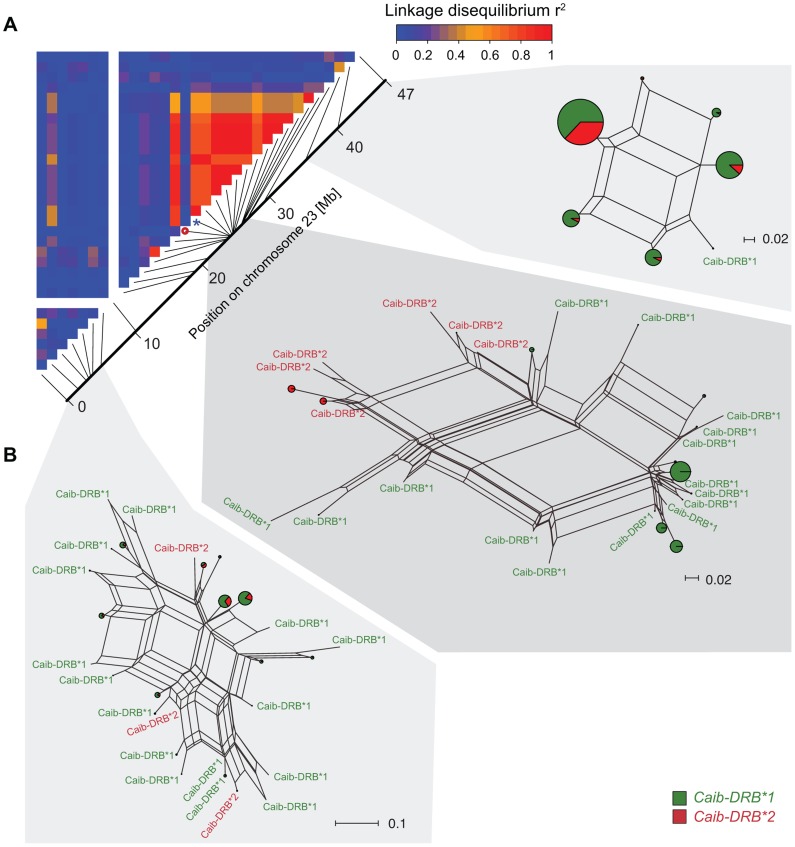
Linkage disequilibria (LD) and haplotype clustering in the region surrounding the MHC of Alpine ibex. (A) LD heatmap (r^2^) of chromosome 23 for the Alpine ibex population Cape au Moine. The color gradient scale represents the range of r^2^ values. Red is used for the highest estimates of linkage disequilibrium. The chromosomal position of SNP16397 (diagnostic for Caib-DRB*2) is indicated by a blue asterisk. The red circle shows a SNP of a low minor allele frequency (0.06) that may explain the low LD. (B) NeigborNet networks were constructed from SNP haplotypes in three regions of chromosome 23. Caib-DRB*1 and Caib-DRB*2 haplotypes are colored in green and red, respectively. Networks are shown separately for three chromosomal sections of similar length comprising 7, 21 and 3 SNPs, respectively.

We extended the SNP genotyping to 95 Alpine ibex individuals (four populations) and 177 domestic goat individuals (six breeds) using the 52 K Illumina Goat SNP Chip [42]. We identified 677 high-quality, polymorphic SNPs among Alpine ibex genome-wide. A total of 35 SNPs were located on chromosome 23 containing the MHC *DRB* locus. We found that allele SNP16397/G was diagnostic for Caib-DRB*2 in all 91 individuals, which were both sequenced at the exon and SNP genotyped (Table S4). We aimed to identify whether haplotypes carrying Caib-DRB*2 were genetically similar in the chromosomal region surrounding the MHC *DRB* locus. As the large chromosomal region was likely to contain recombined haplotypes, we constructed NeighborNet networks [43] based on three different sections of chromosome 23. One section covered 6 Mb of the MHC region containing the *DRB* locus and two sections covered either end of chromosome 23 (Figure 4B). We found that haplotypes containing the diagnostic allele SNP16397/G (associated with Caib-DRB*2) clustered strongly in the section covering the MHC *DRB*. However, we found no such association of haplotypes containing SNP16397/G at either end of the chromosome (Figure 4B). The tight clustering of haplotypes carrying Caib-DRB*2 in the *DRB* MHC region indicates a high relatedness among these haplotypes. This suggests that only few recombination events occurred between haplotypes carrying the Caib-DRB*2 and haplotypes carrying the Caib-DRB*1. If the alleles Caib-DRB*1 and Caib-DRB*2 were maintained as an ancient trans-species polymorphism, no or weak clustering of haplotypes associated with either allele would be expected. The clustering of the haplotypes and the increased expected heterozygosity in the individuals carrying the Caib-DRB*2 allele suggest high levels of linkage disequilibria.

### Population genetic signature of introgression in the MHC *DRB* region

Introgression is expected to generate blocks of linkage disequilibrium due to the recent integration of sequences from another species. We used population and species level data for the analysis of linkage disequilibria in order to control for potential alternative sources of linkage disequilibrium [reviewed in 44]. We identified nearly complete linkage disequilibrium between Caib-DRB*2 and two proximal microsatellite markers (OLADRB1 and OLADRB2) covering a physical distance of 107 to 161 kb in the cattle and sheep genome, respectively (Figure 1C). Complete linkage disequilibrium with Caib-DRB*2 was observed for one of four alleles of the diagnostic marker OLADRB1 (allele OLADRB1/184; n = 156, Figure 1D and Table S4). Allele OLADRB1/184 was nearly completely associated with allele OLADRB2/277 (correspondence between the two markers in 705 out of 707 individuals).

**Figure 5:**
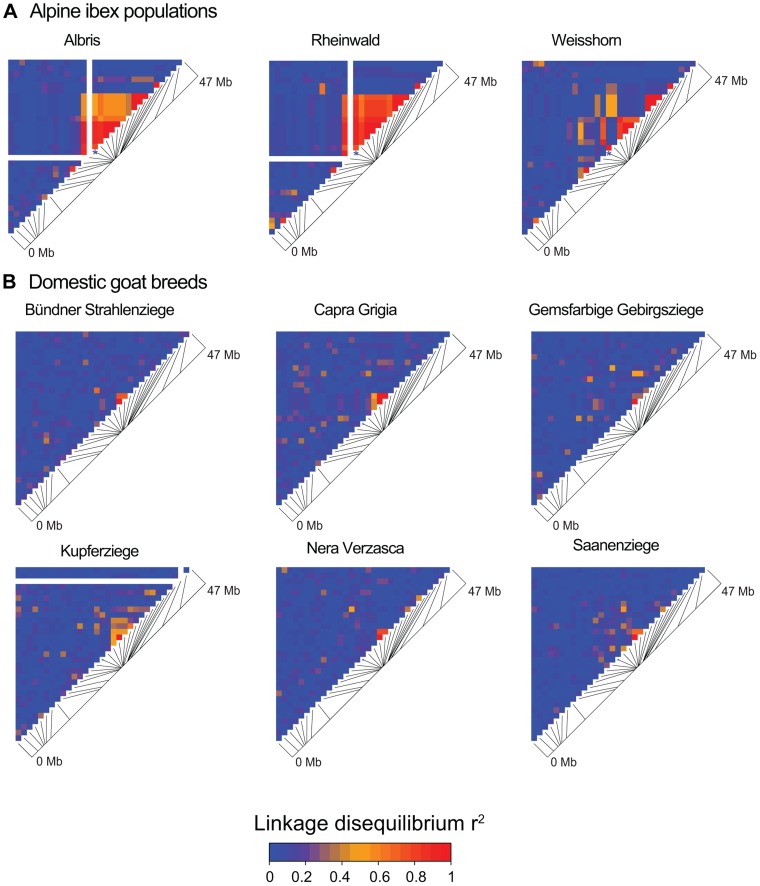
Linkage disequilibrium (LD) heatmaps of chromosome 23 for Alpine ibex populations and domestic goat breeds. (A) The Alpine ibex populations Albris, Rheinwald and Cape au Moine (shown on Figure 4A) have a high frequency of allele Caib-DRB*2 (Table S5). The Weisshorn population has a low frequency of Caib-DRB*2 (Table S5). Populations with a high frequency of Caib-DRB*2 showed larger blocks of strong LD than the Weisshorn population. The asterisks show the position of SNP16397, which was diagnostic for Caib-DRB*2. See Figure S5 for LD heatmaps of all chromosomes of population Albris. (B) In all six domestic goat breeds, LD blocks were generally small.

**Figure 6:**
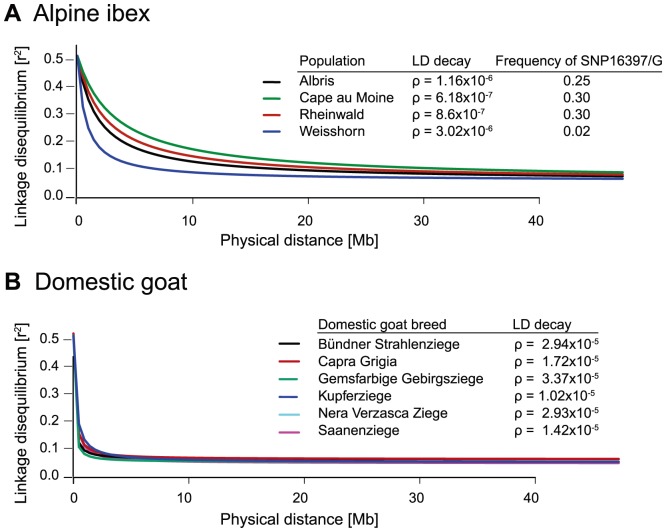
Decay of linkage disequilibria (LD) on chromosome 23. (A) The decay of pairwise linkage disequilibria (r^2^) among each pair of SNPs is shown against the corresponding physical distances. The three Alpine ibex populations Albris, Cape au Moine and Rheinwald showed high Caib-DRB*2 frequencies and a slow decay in LD over distance. Caib-DRB*2 was rare in the Weisshorn population and the LD decay is steeper. Rho (ρ) values provide estimates of the LD decay with higher values indicating steeper decays and shorter LD blocks. For details on Caib-DRB*2 allele frequencies see Table S5. (B) LD decay in six domestic goat breeds. The LD decay is much steeper in domestic goats than in Alpine ibex populations.

Based on data from the 52 K Goat SNP Chip, we calculated linkage disequilibria among SNPs. We found a large block of high linkage disequilibrium containing the MHC *DRB* in Alpine ibex populations with high Caib-DRB*2 allele frequencies (e.g. population Cape au Moine: r^2^ = 0.85 across 2.1 Mb, Figure 4A; Table S5). Two smaller linkage disequilibrium blocks (0.2 and 0.4 Mb with r^2^≥0.85) were observed in the Alpine ibex population with the lowest frequency of the Caib-DRB*2 allele (population Weisshorn, Figure 5A and 6A; Table S5). The smaller blocks of linkage disequilibria in the Weisshorn population may also be explained by a general depletion of genetic diversity at the MHC *DRB*. Compared to Alpine ibex, blocks of linkage disequilibria were much smaller in domestic goats (e.g. breed Capra Grigia: 0.08 Mb with r^2^≥0.85) and linkage disequilibria decreased more steeply with pairwise distance between SNPs (Figures 5B and 6B). Domestic goats showed invariably small linkage disequilibrium blocks. Although introgression is expected to lead to increased linkage disequilibria, population subdivisions, genetic drift, and natural selection may also generate linkage disequilibria [5], [6], [44], [45]. Genetic drift is expected to create randomly distributed regions of high linkage disequilibria among chromosomes and populations [reviewed in 44]. Blocks of strong linkage disequilibria (r^2^>0.8) were generally shorter (0–0.5 Mb) on the chromosomes other than chromosome 23 of Alpine ibex (Figures S4A and S5) and strength of linkage disequilibria was lower for comparable pairwise distances between SNPs (Figure S4B and S4C). Hence, population subdivision and genetic drift are unlikely to explain the large block of high linkage disequilibria at the MHC region in Alpine ibex populations with a high frequency of the Caib-DRB*2 allele.

### The MHC Caib-DRB*2 allele increased rapidly in frequency

Successful introgression of the Caib-DRB*2 allele into Alpine ibex populations would require a significant increase in frequency from the time point of the hybridization event to the extant frequency of the Caib-DRB*2 allele. A significant frequency shift of an allele is expected to leave a footprint of selection in the surrounding chromosomal regions. We aimed to test for evidence of positive selection acting on haplotypes containing Caib-DRB*2 using analyses of extended haplotype homozygosity (EHH). The non-recombined segment of haplotypes containing an allele under positive selection is expected to be much longer and less diverse than haplotypes containing alleles not under selection, because the latter have experienced recombination or mutation events. The EHH measures the length of such conserved haplotypes on both sides of a specified core SNP [46] and has been used to show evidence for selection at the human MHC [5]. For EHH = 1 (maximum) at a certain position, all haplotypes containing the SNP allele of interest are identical up to this position. Introgression events followed by a rapid frequency shift due to drift or selection, are expected to lead to long introgressed haplotypes (i.e. a high EHH over a long distance from the core SNP). The non-recombined part of introgressed haplotypes is expected to be much longer than that of non-introgressed haplotypes.

**Figure 7:**
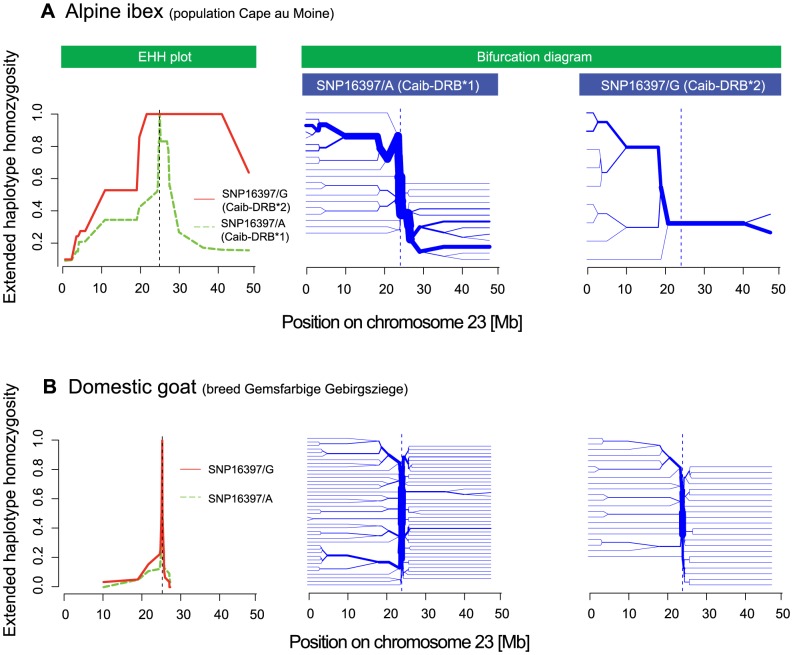
Extended haplotype homozygosity (EHH) plots and bifurcation diagrams. (A) The EHH plot (left panel) of the Alpine ibex population Cape au Moine shows the length of conserved haplotypes on both sides of the SNP diagnostic for Caib-DRB*2 (SNP16397). EHH = 1 indicates that all haplotypes containing the SNP allele (either SNP16397/A or SNP16397/G) are identical up to this position. EHH for SNP16397/G (diagnostic for Caib-DRB*2) is shown in red and the EHH for SNP16397/A (diagnostic for Caib-DRB*1) is shown in green. The bifurcation diagrams (center and right panel) show the branching of haplotypes on both sides of SNP16397. Branches at nodes suggest historical recombination events and the splitting of the haplotype at the node position. The bifurcation diagram for SNP16397/G (diagnostic for Caib-DRB*2) shows much longer haplotypes and fewer branchings at nodes than the bifurcation diagram for SNP16397/A (diagnostic for Caib-DRB*1). (B) The EHH plot and bifurcation diagram for the domestic goat breed Gemsfarbige Gebirgsziege show much shorter haplotypes and more extensive branching at nodes for both SNP alleles. See Figures S6 and S7 for additional Alpine ibex populations and domestic goat breeds.

We found that EHH was substantial around SNP16397 (Figures 7A and S6). The related measure iHH [47], integrated on both sides of the core SNP, showed a similar pattern (data not shown). Alpine ibex haplotypes carrying SNP16397/G (the SNP allele diagnostic for Caib-DRB*2) were substantially longer and were less diverse than haplotypes carrying SNP16397/A (Figure 7A, left panel). Similarly, bifurcation diagrams showed that haplotypes carrying SNP16397/G showed fewer bifurcations that were at a greater distance from the core SNP than haplotypes carrying SNP16397/A. For the six goat breeds, measures of EHH and iHH were generally much lower (Figures 7B and S7). Furthermore, the haplotype diversity associated with either of the two alternative SNP16397 alleles was very similar. Thus, these analyses show that the Caib-DRB*2 allele rapidly increased in frequency and that only few recombination events occurred among haplotypes carrying either Caib-DRB*1 or Caib-DRB*2. The increase in frequency of Caib-DRB*2 could have been caused either by genetic drift and/or by positive selection for individuals carrying the introgressed allele. Positive selection is plausible because the introgression increased the genetic diversity at and around the MHC *DRB*, as evidenced by the fact that individuals lacking Caib-DRB*2 are mainly monomorphic in the region surrounding this locus (Figures 3A and S3).

### Introgression as a source of MHC variation

Using a combination of Sanger and RAD sequencing, SNP chip and microsatellite data we found evidence for introgression at the MHC *DRB* gene in Alpine ibex. The *DRB* exon 2 comprises only two alleles with one allele being identical to an allele found in domestic goats. We found no recombinants between the two highly divergent alleles, while recombinants at this locus were found in several related ungulate species [27], [30], [31]. Alpine ibex homozygous for the goat-type allele showed nearly identical sequences (99.8%) to a breed of domestic goats over 2253 bp of coding and non-coding sequences surrounding the MHC *DRB* exon 2. Evidence of long nearly identical non-coding sequences shared between a domestic goat breed and Alpine ibex shows that introgression rather than ancestral trans-species polymorphism accounts for the MHC *DRB* polymorphism in Alpine ibex.

The chromosomal region in proximity to the MHC *DRB* locus was genetically highly related for the haplotypes carrying the goat-type Caib-DRB*2 allele. We found nearly complete linkage disequilibrium between the MHC locus and two proximal microsatellites covering more than 100 kb. Linkage disequilibria in proximity of the MHC *DRB* locus were strongest in Alpine ibex populations with a high frequency of the Caib-DRB*2 allele. On the contrary, linkage disequilibria were lower in a population with a lower frequency of Caib-DRB*2 and in domestic goat breeds. The extended haplotype homozygosity (EHH) was substantially higher in haplotypes carrying the Caib-DRB*2 than in haplotypes carrying the native Alpine ibex allele, suggesting a substantial increase in allele frequency since the original hybridization event that lead to this introgression. High linkage disequilibria, sequence clustering and increased EHH are consistent with introgression and a selective sweep. We suggest that these signals stem from both the initial introgression event and a subsequent Caib-DRB*2 frequency increase in Alpine ibex populations.

We identified a single introgressed goat allele (Caib-DRB*2) among all sampled populations across Switzerland and in the Gran Paradiso National Park, the founder population of all extant Alpine ibex populations. The most parsimonious explanation for the introgression of Caib-DRB*2 is that the introgression originated from a successful hybridization between domestic goats and Alpine ibex in the Gran Paradiso National Park prior to the reintroduction of Alpine ibex across the Alps. The domestic goat breed Valdostana bred in the vicinity of the Gran Paradiso National Park shows striking phenotypic similarities to Alpine ibex (http://eng.agraria.org). This is indicative of efforts to interbreed Alpine ibex with domestic goats in this region. We suggest that the Caib-DRB*2 allele was introduced to Swiss populations through animals in the captive breeding program at a period of historically low Alpine ibex population sizes. The low extant frequency of Caib-DRB*2 in the population of Gran Paradiso may be explained by the fact that the population passed through a bottleneck after animals were reintroduced to Switzerland [24].

As the domestic goat MHC *DRB* is highly polymorphic, multiple successful introgression events may have introduced different MHC *DRB* alleles into Alpine ibex populations. However, this is unlikely for several reasons. We found no hybrids in the surveyed populations suggesting that hybridization events were rare over the past decades or that hybrids had a lower fitness compared to Alpine ibex. Furthermore, some domestic goat MHC *DRB* alleles may not be of adaptive value for Alpine ibex and, hence, genetic drift may have prevented the successful establishment of such alleles.

Alpine ibex are a genetically impoverished species and were subject to considerable species conservation efforts. Introgression is generally considered a threat for species conservation: small populations of endangered species may be substituted with hybrid individuals and introgression may be maladaptive [48]. Introgression from domesticated species into their wild relatives is of particular concern as shown in the case of the American bison [49]. However, introgression from domestic species was shown to be adaptive in wolves [50] and Soay sheep [18]. MHC introgression from domesticated species may contribute to the genetic rescue of wild relatives.

### Conclusions

We showed that introgression from domestic goats into Alpine ibex generated variation at the previously monomorphic MHC *DRB* locus. MHC *DRB* introgression in Alpine ibex is likely adaptive by broadening the MHC sequence repertoire and thereby conferring an improved immune response. The MHC is a susceptible genomic region for adaptive introgression because balancing selection is expected to favor introgression [17], [19] and alleles are likely to be compatible among species. Introgression may well be an underappreciated mechanism generating the extraordinary genetic diversity at the MHC [16]. Our study supports the view that a broad range of loci under balancing selection may be susceptible to adaptive introgression and will encourage future research to identify unexpected signatures of introgression.

## Materials and Methods

### Sampling

We analyzed 754 Alpine ibex samples from 40 populations across Switzerland, six Swiss wildlife parks and a population from the Gran Paradiso National Park in Italy (Figure 1A). The sample size per population varied between 1–61 individuals (average n = 16, Table S1). Allele frequencies reported in the main text and on Figure 1 are based on populations with n>12. 707 individuals were used to study linkage between the two microsatellites OLADRB1 and OLADRB2 (see below). Samples were collected either as tissue, blood or hair. See [25] for detailed information on the populations and sampling procedures.

### Sanger sequencing of *DRB* exon 2

We based our analyses on a total of 98 exon 2 sequences of the MHC *DRB* class II locus sequenced by Alasaad et al. [28]. In addition to previously published sequences, we sequenced 78 Alpine ibex individuals (Table S4) at the *DRB* exon 2. This locus is homologous to *BoLA-DRB3* and *Ovar-DRB1* of cattle and sheep, respectively (Figure 1C). A nested PCR was performed using the primer pair HL030 (located at the boundary of the first intron and second exon), HL031 and HL032 (both located at the boundary of the second exon and second intron, [27]). The 236 bp PCR product was Sanger sequenced on a 3730 DNA Analyzer (Life Technologies, Inc.). Samples of nine individuals were extracted and sequenced two times independently. Sequences were edited and manually corrected in Geneious, version 6.05 (Biomatters, Inc.). The MHC region of cattle and sheep is homologous to goat chromosome 23 and there is strong colinearity between goat and cattle chromosomes [40]. We verified the sequence homology using BlastN to search the NCBI Genbank database. The closest hits to our sequences were previously published *DRB* exon 2 sequences of Alpine ibex and domestic goats. The MHC class II *DRB* in domestic goats is located on chromosome 23 at 24.9 Mb and is fully contained on scaffold2167 [40]. Correspondence to *BoLA*-*DRB3* and *Ovar*-*DRB1* was verified by BlastN searching the *DRB* exon 2 sequence in the cattle and sheep genomes (Figure 1C).

### Microsatellite genotyping

We genotyped all 754 Alpine ibex individuals at the microsatellite OLADRB1, known to be associated to the MHC region on chromosome 23 [28], [32]. A subset of 707 Alpine ibex individuals was genotyped at the microsatellite locus OLADRB2 located in the same region. OLADRB1 is directly adjacent to the second exon of MHC *DRB* [32]. The forward primer of OLADRB1 overlaps with the primers HL031 and HL032. OLADRB2 (also known as OLADRB, Genbank UniSTS: 251420) has been localized to the BoLA-*DRB2* gene in cattle and is located at 107 kb from exon 2 of MHC *DRB* and OLADRB1 (161 kb in sheep, Figure 1C). See Table S6 for primer sequences and references. PCR conditions for OLADRB2 were as described in [25]. For OLADRB1, we used a reaction volume of 6 µl containing 1.5 µl (3–30 ng) of DNA template, 0.4 µM of both forward and reverse primers and 3 µl Qiagen Multiplex PCR Kit. PCR cycling conditions included an initial denaturation step at 95°C for 15 minutes. Microsatellite quality controls and genotyping procedures were followed according to [25].

### Sanger sequencing of *DRB* regions surrounding exon 2

The *DRB1* gene of domestic sheep was mapped to scaffold2167 of the reference genome of domestic goat. Primers were designed in introns 1 to 4 and 3′ UTR of exon 6 to amplify four loci including partial sequences of introns 1–4 and the complete intron 5 and the 3′ UTR of exon 6. In addition, the loci comprised complete sequences of exons 3, 5 and 6. For the PCR amplification we used a total reaction volume of 25 µl containing 0.5 µM of both forward and reverse primers, 0.2 mM of each dNTPs, 2.5 µl 10× Buffer and Taq Polymerase. PCR cycling conditions included an initial denaturation step at 94°C for 3 minutes, 35 cycles of 30 sec at 94°C, 30 sec at 54°C, 1 min at 72 °C and a final extension of 7 min at 72 °C. See Table S7 for primer sequences. A total of 2253 bp were sequenced according to the protocol described above.

Seven Alpine ibex homozygous for Caib-DRB*1 and seven Alpine ibex homozygous for Caib-DRB*2 were sequenced. Sequenced individuals were chosen according to their genotype at the microsatellite OLADRB1 as described above. Sixteen domestic goat individuals representing five different breeds were sequenced at the locus containing partial sequences of intron 2. Five individuals covering the sequence diversity found at intron 2 were chosen for sequencing at all four loci. Sequences were edited and manually corrected in Geneious, version 6.05. Heterozygous sites were called if homozygote individuals for each allele were identified.

### RAD library preparation

RAD library preparation was performed according to [51] except for the following modifications. A total of 1.35 µg of genomic DNA of each sample was digested with the restriction enzyme *Sbf*1 (New England Biolabs) in a total volume of 50 µl (one hour at 37°C, heat inactivation at 65°C for 20 min, slowly ramp down {<0.1 °C/s}). For the P1 ligation, adapters containing a unique 6 bp barcode (3.5 µl of 100 nM stock prepared according to [51]), 0.5 µl T4 DNA ligase (New England Biolabs; 2,000,000 Weiss Units/ml) and 4.4 µl H_2_O were added to each sample and incubated at room temperature overnight. This was followed by heat inactivation at 65°C for 20 min and slow cool down to room temperature. Samples were pooled before shearing in a COVARIS (Duty Factor: 5%; Peak incidence: 105; Cycles per Burst: 200: Time: 75 s). Size selection (300–700 bp) of the purified DNA fragments was performed using a CALIPER. The excised DNA was purified and blunt-ended (New England Biolabs). 1 µl dATP and 3 µl Klenow enzyme (New England Biolabs) were added (30 min at 37°C) and P2 adapters (1 µl of 10 mM stock) were ligated (0.5 µl rATP of 100 mM; 1 µl of 2,000,000 Weiss U/ml T4 DNA Ligase, New England Biolabs). After purification ligation products were PCR amplified using Phusion High-Fidelity DNA polymerase in a total volume of 120 µl: 50.4 µl H_2_O, 60.0 µl Phusion High Fidelity Master Mix, 2.4 µl primers (10 µM), 4.8 µl library template. Amplification master mixes were divided into 6 separate 20 µl reactions: 98°C 30 s; 14 cycles {98°C 10 s, 65°C 30 s, 72°C 30 s}; final extension for 5′ at 72°C. All purification steps were performed using a MinElute PCR purification kit (Qiagen) according to the manufacturer's recommendations. The library was sequenced on an Illumina HiSeq 2000 platform (100 bp, paired-end).

The FASTX-toolkit was used for P1 barcode splitting (http://hannonlab.cshl.edu/fastx_toolkit/index.html) and Trimmomatic 0.30 (www.usadellab.org/cms/index.php?page=trimmomatic) was used for adapter and quality trimming. Reads were then aligned to the domestic goat reference genome [40] using Bowtie2 2.1.0 [52]. Genotypes were called using UnifiedGenotyper (GATK, version 2.6.5; [53], [54]) and filtered using VariantFiltration and SelectVariants (QD<2.0, MQ<30.0, −12.5>MQRankSum>12.5, FS>40.0, HaplotypeScore>12.0, ReadPosRankSum<−8.000, QUAL>30.0, AN>20).

PGDSpider version 2.0.3.0 [55] was used for data format conversions and to remove genotypes with a genotyping phred-scaled quality score lower than 20. PLINK v. 1.0.7 [56] was used for additional genotype filtering. We required a genotyping rate of SNPs>70% and a minor allele frequency>0.01. PLINK was also used to calculate expected heterozygosity. The R package {ggplot2} was used for data visualization.

### SNP Illumina BeadChip genotyping and filtering

We used a recently developed Illumina InfiniumHD BeadChip [42]. The BeadChip comprises 53'347 SNP markers with known physical locations on the goat genome. We genotyped 96 Alpine ibex individuals from four populations (Albris, Cape au Moine, Rheinwald and Weisshorn; n per population 23–24, Table S5). Populations were chosen in order to cover different frequencies of the putatively introgressed MHC *DRB* allele (Table S1) and the three genetically distinct regions of Alpine ibex [see 25]. In addition, we genotyped 188 domestic goats covering six Swiss breeds: Bündner Strahlenziege, Capra Grigia, Saanenziege, Nera Verzasca, Kupferziege, Gemsfarbene Gebirgsziege (20–49 individuals per breed; see Table S5 for details on sampling).

We used GenomeStudio version 2011.1 for SNP calling and quality filtering. We used PLINK v. 1.0.7 [56] for further locus filtering. Twelve individuals (including one Alpine ibex) were discarded because we required a genotyping rate of at least 90% per individual. Furthermore, SNPs with a genotyping rate lower than 90% (2,817 SNPs) and a minor allele frequency in Alpine ibex below 0.01 (49,832 SNPS) were removed from the data set. Twenty-one additional SNPs were removed because of an observed heterozygosity of one, which may be due to the presence of a duplicated region. A total of 677 SNPs were retained with a genotyping rate of 98% in the remaining individuals. A minor allele frequency of 0.05 (570 SNPs) was applied for the linkage disequilibrium analysis, resulting in a genotyping rate of 99.6%. PGDSpider version 2.0.3.0 [55] was used for data format conversions.

### Phylogenetic and population genetic analyses

We constructed haplotype networks of phased SNP data (fastPHASE version 1.4.0, [57]) based on the Neighbor-Net algorithm implemented in Splitstree 4.13.1 [43]. The networks were based on the uncorrected *p* distance using concatenated SNP loci. Pairwise linkage disequilibrium (LD; r^2^) estimates among SNPs were obtained using the R package *{genetics}*. Heatmaps of pairwise LD estimates were produced using the function LDheatmap in R package *{LDheatmap}*. In order to investigate the decay of LD over distance, a regression of r^2^ values against pairwise distance was plotted (Figure 6). The expected values of r^2^ were computed using non linear least squares (*nls* in R) as shown in the following function by [58].
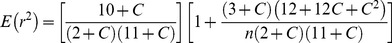



Where n is the number of haplotypes, *C* = ρ * distance[bp] and ρ = 4N_e_r (population recombination rate). All LD analyses were performed for each Alpine ibex population and domestic goat breed separately.

The software STRUCTURE [39] was used in order to search for recent hybrids. See figure legends S1 and S2 for details on parameter values.

Measures of the extended haplotype homozygosity (EHH, [46], Figures 7, S6, S7) were calculated from phased data (fastPHASE version 1.4.0, [57]) using the R package *{rehh}* [59]. The same package was used for the bifurcation diagrams. We used physical distances for the EHH analyses and refrained from performing statistical tests, as we did not have access to genotyped Alpine ibex families and, hence, estimates of genetic distances.

### MHC *DRB* sequence diversity analyses

For the comparisons of MHC *DRB* exon 2 sequences among species of the subfamily Caprinae, a BlastN of Caib-DRB*1 was performed in Geneious version 6.05 (Biomatters, Inc.). Sequences were aligned using MAFFT v7.023b [60]. We used the R function *dist.dna {ape}* (model = “raw”, proportion of sites that differ between each pair of sequences, no mutation model) to calculate genetic distances between all pairs of sequences. As most published sequences were shorter than our sequenced *DRB* exon 2 fragment, we used a universal 227 bp sequence length to calculate percent identities. We used R to identify sequences shared among species. This comparison was done both for 236 bp (112 sequences unique within species) and 227 bp (332 sequences unique within species).

We tested for evidence of recombination at the MHC *DRB* using the Φw-statistic developed by [41] implemented in Splitstree4 [43], We used the default window size of 100 and k = 5 (Alpine ibex) and k = 3 (domestic goats).

## Supporting Information

Figure S1 Genetic clustering of domestic goat and Alpine ibex based on microsatellites. The software STRUCTURE was used to identify the group assignment of 1781 Alpine ibex (yellow) and 182 domestic goats (blue) genotyped at 30 neutral microsatellites [this study and 25]. We also included three known recent F1 hybrids (see zoomed section). Except for the three known F1 hybrids, we did not detect any other recent hybrids (q<0.02). The parameters for the STRUCTURE analysis were as follows: K = 2, length of burnin period: 20'000; number of MCMC replicates after burnin: 80'000; ancestry model: admixture; allele frequency model: allele frequencies correlated among populations.
(EPS)

Figure S2 Genetic clustering of domestic goat and Alpine ibex based on SNP chip data. The software STRUCTURE was used to identify the group assignment of 95 Alpine ibex (yellow) and 177 domestic goats (blue) based on 546 genotyped SNPs. STRUCTURE assumes that markers are either at linkage equilibrium or weakly linked. Therefore, we pruned out loci in strong linkage disequilibrium within species using the option “—indep 50 5 2″ in PLINK (window size in number of SNPs: 50; number of SNPs to shift the window at each step: 5; variance inflation factor criterion (VIF) threshold: 2). VIF is calculated as VIF = 1/(1−R^2^), where R^2^ is the multiple correlation coefficient for a SNP being regressed on all other SNPs in a certain window simultaneously (in our analyses a window of 50 SNPs spans the entire chromosome). A VIF of 1 implies that the SNP is completely independent of all other SNPs within the window. We did not detect any recent hybrids (q<0.08). The parameters for the STRUCTURE analysis were as follows: K = 2, length of burn-in period: 20'000; number of MCMC replicates after burnin: 80'000; ancestry model: admixture; allele frequency model: allele frequencies correlated among populations. STRUCTURE results were consistent when running the analyses either using all 677 SNPs or using different models (admixture model or the linkage model).
(EPS)

Figure S3 Increased heterozygosity in a 750 kb region surrounding the MHC *DRB* in Alpine ibex carrying Caib-DRB*2. (A) Sliding window of expected heterozygosity for nine domestic goat individuals genotyped using RAD sequencing (window size: 500 kb). (B) Sliding window of expected heterozygosity for 15 Alpine ibex individuals homozygous for Caib-DRB*1 (blue) and 15 Alpine ibex individuals carrying Caib-DRB*2 (green). (C) Number of RAD sequencing SNPs per 100 kb polymorphic (minor allele frequency = 0.01) in domestic goats (light blue) and Alpine ibex (dark blue).
(EPS)

Figure S4 Linkage disequilibrium (LD) block size of chromosome 23 compared to the other chromosomes. (A) Distribution of different LD strength classes for all SNP pairs up to a distance of 6 Mb. We included all three populations with a high frequency of Caib-DRB*2 (Albris, Cape au Moine, Rheinwald) and all chromosomes except the sex chromosome (chromosome 30). The distribution of LD is shown for three different classes: strong (r^2^>0.8), moderate (0.5<r^2^<0.8) and weak (0.2<r^2^<0.5) LD. LD blocks on chromosome 23 (red) were generally longer than on the other chromosomes (blue) for the same class of LD strength. (B) Global distribution of LD strength classes on chromosome 23 (red) compared to all other chromosomes (blue). In order to avoid potential effects of variable marker densities among chromosomes, we restricted the plot to SNP pairs of a distance below 1 Mb. High measures of LD were more frequent on chromosome 23 than on other chromosomes. (C) Boxplots of pairwise distances among SNPs for the different chromosomes. Only SNP pairs of a distance below 1 Mb are shown as in panel B.
(EPS)

Figure S5 Linkage disequilibrium (LD) heatmaps for all chromosomes of the Albris population. The color gradient scale represents the range of r^2^ values. Red is used for the highest estimates of linkage disequilibria. The signature of strong linkage disequilibria observed on chromosome 23 was exceptional when compared with other chromosomes. However, the SNP density in the region surrounding the MHC *DRB* was high compared to other chromosomal regions. To control for potential effects of marker density, we show a comparison of LD block size between chromosome 23 and the other chromosomes and an overview of marker densities in Figure S4.
(EPS)

Figure S6 Extended haplotype homozygosity (EHH) plots and bifurcation diagrams for Alpine ibex populations. The EHH plot (left panel) of the Alpine ibex populations Albris, Cape au Moine and Rheinwald shows the length of conserved haplotypes on both sides of the SNP diagnostic for Caib-DRB*2 (SNP16397). An EHH = 1 indicates that all haplotypes containing the SNP allele (either SNP16397/A or SNP16397/G) are identical up to this position. EHH for SNP16397/G (diagnostic for Caib-DRB*2) is shown in red and the EHH for SNP16397/A (diagnostic for Caib-DRB*1) is shown in green. The bifurcation diagrams (center and right panel) show the branching of haplotypes on both sides of SNP16397. Branches at nodes suggest historical recombination events and the splitting of the haplotype at the node position. The bifurcation diagram for SNP16397/G (diagnostic for Caib-DRB*2) shows much longer haplotypes and fewer branchings at nodes than the bifurcation diagram for SNP16397/A (diagnostic for Caib-DRB*1). EHH and bifurcation analyses were not possible for the Weisshorn population, because there was only one haplotype containing SNP16397/G. See also Table S5.
(EPS)

Figure S7 Extended haplotype homozygosity (EHH) plots and bifurcation diagrams for domestic goat breeds. The EHH plot and bifurcation diagram for six domestic goat breeds show much shorter haplotypes and more extensive branching at nodes for both SNP alleles. See Figure S6 for further details on EHH and bifurcation diagrams.
(EPS)

Table S1 Allele frequencies of Caib-DRB*2 estimated using the microsatellite OLADRB1 in Alpine ibex populations.
(DOCX)

Table S2 Number of MHC *DRB* exon 2 alleles found in Caprinae species. We report the mean pairwise sequence distances based on 236 bp sequence length. Numbers in parenthesis show pairwise distances for 227 bp sequence lengths. Distances are based on the percentage of sites that differ between each pair of sequences.
(DOCX)

Table S3 NCBI accession numbers and corresponding species names for MHC *DRB* exon 2 sequences that are shared among species pairs. Comparisons are based on 227 bp sequence lengths. References documenting the occurrence of hybrids among the species pairs are shown if available.
(DOCX)

Table S4 *Caib-DRB* genotypes identified through Sanger sequencing, SNP chip (SNP16397) and microsatellite genotyping (OLADRB1) for all individuals sequenced at the *DRB* exon 2. Column rep indicates samples that were sequenced twice. Column RAD indicates samples that were used for RAD sequencing. MHC *DRB* exon 2 and microsatellite genotypes are combined from [28] and this study.
(DOCX)

Table S5 Sample sizes of Alpine ibex populations and domestic goat breeds included in the SNP genotyping. The allele frequencies at SNP16397 (diagnostic for Caib-DRB*2) are shown for each population and breed, respectively.
(DOCX)

Table S6 Microsatellites linked to the MHC *DRB* locus. Names in italic and brackets are synonyms for the microsatellite names used in the text. ^1)^ Chromosomal location and gene region; ^2)^ T_a_: annealing temperature; ^3)^ Number of cycles.
(DOCX)

Table S7 Primer sequences used for amplifying four loci of the MHC *DRB* gene of Alpine ibex and domestic goat. Locus “Intron 4 - Exon 6” corresponds to the locus spanning partial intron 4, exon 5, intron 5, exon 6, 3′ UTR and was amplified using two primer pairs.
(DOCX)

Text S1 MHC *DRB* sequence alignment. Partial intron 1.
(DOC)

Text S2 MHC *DRB* sequence alignment. Partial intron 2.
(DOC)

Text S3 MHC *DRB* sequence alignment. Partial intron 2, exon 3, partial intron 3.
(DOC)

Text S4 MHC *DRB* sequence alignment. Partial intron 4, exon 5, intron 5, exon 6, 3′ UTR.
(DOC)
