# Supplementary material for: Introgression from Domestic Goat Generated Variation at the Major Histocompatibility Complex of Alpine Ibex
Source: PLoS Genet. 2014 Jun 19;10(6):e1004438. doi: 10.1371/journal.pgen.1004438 (PMC4063738; doi:10.1371/journal.pgen.1004438)
Supplement: Table S1 — Allele frequencies of Caib-DRB*2 estimated using the microsatellite OLADRB1 in Alpine ibex populations. (DOCX) [file pgen.1004438.s008.docx]

## **Table S1:** Allele frequencies of Caib-DRB*2 estimated using the microsatellite OLADRB1 in Alpine ibex populations.

|  |  | Microsatellite alleles frequencies | | | |
| --- | --- | --- | --- | --- | --- |
|  |  | (alleles identified by their length in bp) | | | |
| **Swiss populations** | n individuals | 170 | 174 | 178 | 184 (*Caib-DRB*2*) |
| Adula-Vial | 37 | 0.00 | 0.64 | 0.27 | 0.10 |
| Albris * | 39 | 0.01 | 0.60 | 0.24 | 0.14 |
| Aletsch-Bietschhorn | 25 | 0.44 | 0.56 | 0.00 | 0.00 |
| Arolla | 12 | 0.33 | 0.67 | 0.00 | 0.00 |
| Brienzer-Rothorn | 27 | 0.06 | 0.72 | 0.22 | 0.00 |
| Calanda | 19 | 0.00 | 0.50 | 0.26 | 0.24 |
| Cape au Moine * | 21 | 0.05 | 0.48 | 0.17 | 0.31 |
| Crap da Flem | 5 | 0.20 | 0.60 | 0.10 | 0.10 |
| Dents du Midi | 10 | 0.35 | 0.65 | 0.00 | 0.00 |
| Toedi | 4 | 0.00 | 0.25 | 0.50 | 0.25 |
| Fergen Seetal | 4 | 0.00 | 0.25 | 0.63 | 0.13 |
| Ferret | 2 | 0.25 | 0.75 | 0.00 | 0.00 |
| Flueela | 20 | 0.00 | 0.65 | 0.28 | 0.08 |
| Gornergrat | 4 | 0.50 | 0.50 | 0.00 | 0.00 |
| Graue Hoerner | 30 | 0.00 | 0.63 | 0.25 | 0.12 |
| Gross Lohner | 9 | 0.56 | 0.33 | 0.11 | 0.00 |
| Hochwang | 25 | 0.00 | 0.52 | 0.26 | 0.22 |
| Julier Nord | 4 | 0.00 | 0.38 | 0.38 | 0.25 |
| Julier Sued | 14 | 0.04 | 0.25 | 0.61 | 0.11 |
| Macun | 22 | 0.00 | 0.41 | 0.32 | 0.27 |
| Mischabel | 29 | 0.35 | 0.66 | 0.00 | 0.00 |
| Muveran | 12 | 0.21 | 0.67 | 0.13 | 0.00 |
| Nufenen | 11 | 0.73 | 0.27 | 0.00 | 0.00 |
| Oberalp | 3 | 0.00 | 0.33 | 0.33 | 0.33 |
| Pierreuse-Gummfluh | 20 | 0.13 | 0.60 | 0.20 | 0.08 |
| Pleureur | 14 | 0.57 | 0.43 | 0.00 | 0.00 |
| Rheinwald * | 30 | 0.00 | 0.45 | 0.30 | 0.25 |
| Rothorn-Weissfluh | 25 | 0.00 | 0.52 | 0.24 | 0.24 |
| Schwarzmoench | 27 | 0.09 | 0.76 | 0.15 | 0.00 |
| Tanay | 13 | 0.46 | 0.54 | 0.00 | 0.00 |
| Tasna | 8 | 0.00 | 0.44 | 0.50 | 0.06 |
| Terza | 1 | 0.00 | 1.00 | 0.00 | 0.00 |
| Umbrail | 23 | 0.15 | 0.44 | 0.24 | 0.17 |
| Val Bever | 20 | 0.00 | 0.65 | 0.25 | 0.10 |
| Vereina | 5 | 0.00 | 0.70 | 0.20 | 0.10 |
| Weisshorn * | 9 | 0.17 | 0.78 | 0.00 | 0.06 |
| Weissmies | 47 | 0.29 | 0.71 | 0.00 | 0.00 |
| Wetterhorn | 18 | 0.69 | 0.31 | 0.00 | 0.00 |
| Wittenberg | 20 | 0.13 | 0.53 | 0.15 | 0.20 |
|  |  |  |  |  |  |
| **Gran Paradiso** | 61 | 0.44 | 0.53 | 0.00 | 0.03 |
|  |  |  |  |  |  |
| **Wildlife Parks (zoos)** |  |  |  |  |  |
| WP.Daehlh | 3 | 0.00 | 1.00 | 0.00 | 0.00 |
| WP.Goldau | 8 | 0.00 | 0.75 | 0.00 | 0.25 |
| WP.Harder | 2 | 0.00 | 1.00 | 0.00 | 0.00 |
| WP.Langenb | 4 | 0.00 | 0.63 | 0.00 | 0.38 |
| WP.Roggenh | 1 | 0.50 | 0.50 | 0.00 | 0.00 |
| WP.PetPau | 7 | 0.00 | 0.79 | 0.21 | 0.00 |
|  |  |  |  |  |  |
| Overall | 754 | 0.17 | 0.57 | 0.16 | 0.10 |

* populations for which SNP genotypes are available.
