# Supplementary material for: Introgression from Domestic Goat Generated Variation at the Major Histocompatibility Complex of Alpine Ibex
Source: PLoS Genet. 2014 Jun 19;10(6):e1004438. doi: 10.1371/journal.pgen.1004438 (PMC4063738; doi:10.1371/journal.pgen.1004438)
Supplement: Table S2 — Number of MHC DRB exon 2 alleles found in Caprinae species. We report the mean pairwise sequence distances based on 236 bp sequence length. Numbers in parenthesis show pairwise distances for 227 bp sequence lengths. Distances are based on the percentage of sites that differ between each pair of sequences. (DOCX) [file pgen.1004438.s009.docx]

## **Table S2:** Number of MHC DRB exon 2 alleles found in Caprinae species. We report the mean pairwise sequence distances based on 236 bp sequence length. Numbers in parenthesis show pairwise distances for 227 bp sequence lengths. Distances are based on the percentage of sites that differ between each pair of sequences.

| Common name | Scientific name | Number of alleles | Mean distance | 95% Confidence interval |
| --- | --- | --- | --- | --- |
| Domestic goat | *Capra hircus* | 14 (44) | 8.33 (8.60) | 1.27;14.41 (1.76;14.98) |
| Spanish ibex | *Capra pyrenaica* | 2 (6) | 14.83 (10.37) | NA;NA (0.88;14.98) |
| Domestic sheep | *Ovis aries* | 73 (184) | 6.79 (7.42) | 1.69;14.83 (2.20;15.86) |
| Thinhorn sheep | *Ovis dalli* | NA (15) | NA (6.59) | NA;NA (0.89;14.22) |
| Himalayan Thar | *Hemitragus jemlahicus* | 2 (5) | 4.24 (7.62) | NA;NA (4.41;9.25) |
| Chamois | *Rupicapra rupicapra* | 9 (40) | 3.15 (3.91) | 0.85;5.51 (0.88;6.61) |
| Bighorn sheep | *Ovis canadensis* | 7 (21) | 8.90 (8.07) | 0.42;14.83 (0.44;14.98) |
| Mountain goat | *Oreamnos americanus* | NA (1) | NA (NA) | NA;NA (NA;NA) |
| Pyrenean chamois | *Rupicapra pyrenaica* | 3 (13) | 4.10 (4.59) | 2.97;5.51 (1.32;7.05) |
| Musk ox | *Ovibos moschatus* | NA (1) | NA (NA) | NA;NA (NA;NA) |
| Alpine ibex | *Capra ibex ibex* | 2 (2) | 13.14 (13.22) | NA;NA (NA;NA) |
