# Supplementary material for: Introgression from Domestic Goat Generated Variation at the Major Histocompatibility Complex of Alpine Ibex
Source: PLoS Genet. 2014 Jun 19;10(6):e1004438. doi: 10.1371/journal.pgen.1004438 (PMC4063738; doi:10.1371/journal.pgen.1004438)
Supplement: Table S3 — NCBI accession numbers and corresponding species names for MHC DRB exon 2 sequences that are shared among species pairs. Comparisons are based on 227 bp sequence lengths. References documenting the occurrence of hybrids among the species pairs are shown if available. (DOCX) [file pgen.1004438.s010.docx]

**Table S3:** NCBI accession numbers and corresponding species names for MHC *DRB* exon 2 sequences that are shared among species pairs. Comparisons are based on 227 bp sequence lengths. References documenting the occurrence of hybrids among the species pairs are shown if available.

| Species pairs sharing *DRB* exon 2 alleles  (NCBI accession numbers) | | References documenting hybridization | |
| --- | --- | --- | --- |
| *Capra aegagrus* (AB008361, Z92720) | *Capra ibex ibex*  (this study) | [1,2*] | |
| *Capra aegagrus*  (Z92724) | *Capra pyrenaica*  (AF461693) | [3] | |
| *Ovis aries*  (FM209041, AF036559) | *Ovis dalli*  (AJ920403) | *O. canadensis* (close relative of *O. dalli*) x *O. aries* [4] | |
| *Capra aegagrus*  (Z92725) ° | *Ovis aries*  (JF898317) ° | [5*,6*,7] | |
| *Ovis canadensis*  (JN081875, AF324841, AF324843) | *Ovis dalli*  (AJ920401) | [8] | |
| *Ovis canadensis*  (JN081871, AF324857, AJ968652) | *Ovis dalli*  (AJ920399) | [8] | |
| *Ovis canadensis*  (JN081870, AF324856) | *Ovis dalli*  (AJ920400) | [8] | |
| *Ovis canadensis*  (AF324859) | *Ovis dalli*  (AJ920397) | [8] | |
| *Rupicapra pyrenaica*  (AY212156) | *Rupicapra rupicapra*  (EU887504) | [9,10] | |
| *Rupicapra pyrenaica*  (AY898755) | *Rupicapra rupicapra*  (AY368451) | [9,10] | |
| *Rupicapra pyrenaica*  (AY212152) | *Rupicapra rupicapra*  (EU887507) | [9,10] | |
| *Rupicapra pyrenaica*  (AY898754) | *Rupicapra rupicapra*  (EU887493) | [9,10] | |
| *Rupicapra pyrenaica*  (AY212150) | *Rupicapra rupicapra*  (AY368437) | [9,10] | |
| ° These two sequences are not identical across the published 237 bp | | |  |

* references showing that hybrids of the two species are fertile

# *References*

1. Couturier (1962) Hybrides entre le bouquetin des alpes et la chevre domestique, et hybrides entre les differentes formes du genre *Capra*.

2. Giacometti M, Roganti R, de Tann D, Stahlberger-Saitbekova N, Obexer-Ruff G (2004) Alpine ibex *Capra ibex ibex* x domestic goat *C. aegagrus domestica* hybrids in a restricted area of southern Switzerland. Wildlife Biol 10: 137–143.

3. Alasaad S, Fickel J, Rossi L, Sarasa M, Benã-Tez-Camacho B, et al. (2012) Applicability of major histocompatibility complex *DRB1* alleles as markers to detect vertebrate hybridization: a case study from Iberian ibex × domestic goat in southern Spain. Acta Vet Scand 54: 56. doi:10.1186/1751-0147-54-56.

4. Young SP, Manville RH (1960) Records of bighorn hybrids. J Mammal 41: 523–525.

5. Cribiu EP, Matejka M, Denis B, Malher X (1988) Etude chromosomique d'un hybride chèvre x mouton fertile. Genet Sel Evol 20: 379–386.

6. Tucker EM, Denis B, Kilgour L (1989) Blood genetic marker studies of a sheep-goat hybrid and its back-cross offspring. Anim Genet 20: 179–186.

7. Mine OM, Kedikilwe K, Ndebele RT, Nsoso SJ (2000) Sheep-goat hybrid born under natural conditions. Small Ruminant Res 37: 141–145.

8. Loehr J, Carey J, Ylönen H, Suhonen J (2008) Coat darkness is associated with social dominance and mating behaviour in a mountain sheep hybrid lineage. Anim Behav 76: 1545–1553. doi:10.1016/j.anbehav.2008.07.012.

9. Rodríguez F, Hammer S, Pérez T, Suchentrunk F, Lorenzini R, et al. (2009) Cytochrome b phylogeography of chamois (*Rupicapra* spp.). Population contractions, expansions and hybridizations governed the diversification of the genus. J Hered 100: 47–55. doi:10.1093/jhered/esn074.

10. Rodríguez F, Pérez T, Hammer SE, Albornoz J, Domínguez A (2010) Integrating phylogeographic patterns of microsatellite and mtDNA divergence to infer the evolutionary history of chamois (genus *Rupicapra*). Bmc Evol Biol 10: 222. doi:10.1186/1471-2148-10-222.
