# Supplementary material for: Introgression from Domestic Goat Generated Variation at the Major Histocompatibility Complex of Alpine Ibex
Source: PLoS Genet. 2014 Jun 19;10(6):e1004438. doi: 10.1371/journal.pgen.1004438 (PMC4063738; doi:10.1371/journal.pgen.1004438)
Supplement: Table S4 — Caib-DRB genotypes identified through Sanger sequencing, SNP chip (SNP16397) and microsatellite genotyping (OLADRB1) for all individuals sequenced at the DRB exon 2. Column rep indicates samples that were sequenced twice. Column RAD indicates samples that were used for RAD sequencing. MHC DRB exon 2 and microsatellite genotypes are combined from [28] and this study. (DOCX) [file pgen.1004438.s011.docx]

**Table S4:** *Caib-DRB g*enotypes identified through Sanger sequencing, SNP chip (SNP16397) and microsatellite genotyping (OLADRB1) for all individuals sequenced at the *DRB* exon 2. Column rep indicates samples that were sequenced twice. Column RAD indicates samples that were used for RAD sequencing. MHC *DRB* exon 2 and microsatellite genotypes are combined from Alasaad et al. 2012 and this study.

| ID | Population | rep | MHC *DRB* exon 2 | microsatellite | SNP | RAD |
| --- | --- | --- | --- | --- | --- | --- |
| BE0113 | Brienzer-Rothorn |  | **Caib-DRB*1 ; Caib-DRB*1** | **174 ; 178** |  |  |
| BE0114 | Brienzer-Rothorn |  | **Caib-DRB*1 ; Caib-DRB*1** | **174 ; 174** |  |  |
| BE0115 | Brienzer-Rothorn |  | **Caib-DRB*1 ; Caib-DRB*1** | **174 ; 178** |  |  |
| BE0116 | Brienzer-Rothorn |  | **Caib-DRB*1 ; Caib-DRB*1** | **174 ; 174** |  |  |
| BE0117 | Brienzer-Rothorn |  | **Caib-DRB*1 ; Caib-DRB*1** | **174 ; 174** |  |  |
| BE0118 | Augstmatthorn |  | **Caib-DRB*1 ; Caib-DRB*1** | **174 ; 174** |  |  |
| BE0119 | Schwarzmoench |  | **Caib-DRB*1 ; Caib-DRB*1** | **174 ; 178** |  |  |
| BE0120 | Schwarzmoench |  | **Caib-DRB*1 ; Caib-DRB*1** | **174 ; 174** |  |  |
| BE0125 | Schwarzmoench |  | **Caib-DRB*1 ; Caib-DRB*1** | **170 ; 170** |  |  |
| BE0126 | Schwarzmoench |  | **Caib-DRB*1 ; Caib-DRB*1** | **174 ; 174** |  |  |
| BE0127 | Schwarzmoench |  | **Caib-DRB*1 ; Caib-DRB*1** | **174 ; 174** |  |  |
| BE0150 | Wittenberg |  | **Caib-DRB*1** ; **Caib-DRB*2** | **170 ; 184** |  |  |
| BE0151 | Wittenberg |  | **Caib-DRB*1** ; **Caib-DRB*2** | **174 ; 184** |  |  |
| BE0152 | Pierreuse-Gummfluh |  | **Caib-DRB*1** ; **Caib-DRB*2** | **174 ; 184** |  |  |
| BE0154 | Pierreuse-Gummfluh |  | **Caib-DRB*1** ; **Caib-DRB*2** | **174 ; 184** |  |  |
| GP1206 | Gran Paradiso |  | **Caib-DRB*1 ; Caib-DRB*1** | **170 ; 170** |  |  |
| GP1706 | Gran Paradiso |  | **Caib-DRB*1 ; Caib-DRB*1** | **174 ; 174** |  |  |
| GP4606 | Gran Paradiso |  | **Caib-DRB*1 ; Caib-DRB*1** | **174 ; 174** |  |  |
| GPC01F | Gran Paradiso |  | **Caib-DRB*1 ; Caib-DRB*1** | **174 ; 174** |  |  |
| GPO01E | Gran Paradiso |  | **Caib-DRB*1 ; Caib-DRB*1** | **170 ; 170** |  |  |
| GPO05E | Gran Paradiso |  | **Caib-DRB*1 ; Caib-DRB*1** | **174 ; 174** |  |  |
| GPO13F | Gran Paradiso |  | **Caib-DRB*1 ; Caib-DRB*1** | **170 ; 170** |  |  |
| GPR21 | Gran Paradiso |  | **Caib-DRB*1** ; **Caib-DRB*2** | **170 ; 184** |  |  |
| GPR22 | Gran Paradiso |  | **Caib-DRB*1** ; **Caib-DRB*2** | **170 ; 184** |  |  |
| GPV11E | Gran Paradiso |  | **Caib-DRB*1 ; Caib-DRB*1** | **170 ; 170** |  |  |
| GPV12E | Gran Paradiso |  | **Caib-DRB*1 ; Caib-DRB*1** | **174 ; 174** |  |  |
| GR0001 | Albris |  | **Caib-DRB*1** ; **Caib-DRB*2** | **178 ; 184** | **A ; G** | ***** |
| GR0002 | Albris |  | **Caib-DRB*1 ; Caib-DRB*1** | **174 ; 174** | **A ; A** |  |
| GR0003 | Albris |  | **Caib-DRB*1 ; Caib-DRB*1** | **174 ; 174** | **A ; A** |  |
| GR0005 | Albris |  | **Caib-DRB*1** ; **Caib-DRB*2** | **174 ; 184** | **A ; G** | ***** |
| GR0006 | Albris |  | **Caib-DRB*1 ; Caib-DRB*1** | **174 ; 178** | NaN |  |
| GR0008 | Albris |  | **Caib-DRB*1 ; Caib-DRB*1** | **174 ; 174** | **A ; A** | ***** |
| GR0009 | Albris |  | **Caib-DRB*1** ; **Caib-DRB*2** | **174 ; 184** | **A ; G** | ***** |
| GR0010 | Albris |  | **Caib-DRB*1 ; Caib-DRB*1** | **174 ; 174** | **A ; A** | ***** |
| GR0113 | Albris |  | **Caib-DRB*1 ; Caib-DRB*1** | **174 ; 174** | **A ; A** | ***** |
| GR0129 | Albris |  | **Caib-DRB*1 ; Caib-DRB*1** | **174 ; 178** | **A ; A** |  |
| GR0139 | Rheinwald |  | **Caib-DRB*1** ; **Caib-DRB*2** | **174 ; 184** | **A ; G** |  |
| GR0140 | Rheinwald | ***** | **Caib-DRB*2 ; Caib-DRB*2** | **184 ; 184** | **G ; G** |  |
| GR0141 | Rheinwald |  | **Caib-DRB*1** ; **Caib-DRB*2** | **178 ; 184** | **A ; G** |  |
| GR0147 | Albris |  | **Caib-DRB*1** ; **Caib-DRB*2** | **174 ; 184** | **A ; G** |  |
| GR0150 | Albris |  | **Caib-DRB*1 ; Caib-DRB*1** | **174 ; 174** | **A ; A** |  |
| GR0197 | Julier Sued |  | **Caib-DRB*1 ; Caib-DRB*1** | **178 ; 178** |  |  |
| GR0199 | Albris |  | **Caib-DRB*1** ; **Caib-DRB*2** | **174 ; 184** | **A ; G** |  |
| GR0201 | Albris |  | **Caib-DRB*2 ; Caib-DRB*2** | **184 ; 184** | **G ; G** | ***** |
| GR0227 | Rheinwald |  | **Caib-DRB*1 ; Caib-DRB*1** | **174 ; 178** | **A ; A** |  |
| GR0235 | Rheinwald | ***** | **Caib-DRB*1** ; **Caib-DRB*2** | **178 ; 184** | **A ; G** |  |
| GR0247 | Albris |  | **Caib-DRB*1 ; Caib-DRB*1** | **174 ; 174** | NaN |  |
| GR0308 | Rheinwald |  | **Caib-DRB*1 ; Caib-DRB*1** | **174 ; 174** | NaN |  |
| GR0309 | Rheinwald |  | **Caib-DRB*1 ; Caib-DRB*1** | **174 ; 178** | **A ; A** |  |
| GR0310 | Rheinwald | ***** | **Caib-DRB*2 ; Caib-DRB*2** | **184 ; 184** | **G ; G** |  |
| GR0311 | Rheinwald |  | **Caib-DRB*1 ; Caib-DRB*1** | **174 ; 174** | **A ; A** |  |
| GR0322 | Albris |  | **Caib-DRB*1 ; Caib-DRB*1** | **178 ; 178** |  |  |
| GR0323 | Albris |  | **Caib-DRB*1** ; **Caib-DRB*2** | **174 ; 184** | **A ; G** |  |
| GR0379 | Albris |  | **Caib-DRB*1 ; Caib-DRB*1** | **174 ; 174** | **A ; A** | ***** |
| GR0381 | Albris |  | **Caib-DRB*1 ; Caib-DRB*1** | **174 ; 174** | **A ; A** |  |
| GR0383 | Albris |  | **Caib-DRB*1** ; **Caib-DRB*2** | **174 ; 184** | **A ; G** |  |
| GR0384 | Albris |  | **Caib-DRB*1 ; Caib-DRB*1** | **174 ; 178** | **A ; A** |  |
| GR0442 | Albris |  | **Caib-DRB*1 ; Caib-DRB*1** | **174 ; 178** | **A ; A** |  |
| GR0452 | Duess.Toed |  | **Caib-DRB*1 ; Caib-DRB*1** | **178 ; 178** |  |  |
| GR0608 | Adula-Vial |  | **Caib-DRB*1 ; Caib-DRB*1** | **174 ; 174** |  |  |
| GR0613 | Oberalp |  | **Caib-DRB*1 ; Caib-DRB*1** | **174 ; 178** |  |  |
| GR0616 | Oberalp |  | **Caib-DRB*2 ; Caib-DRB*2** | **184 ; 184** |  |  |
| GR0698 | Rheinwald |  | **Caib-DRB*1 ; Caib-DRB*1** | **174 ; 174** | **A ; A** |  |
| GR0700 | Rheinwald |  | **Caib-DRB*1** ; **Caib-DRB*2** | **178 ; 184** |  |  |
| GR0701 | Adula-Vial |  | **Caib-DRB*1 ; Caib-DRB*1** | **178 ; 178** |  |  |
| GR0702 | Adula-Vial |  | **Caib-DRB*1 ; Caib-DRB*1** | **174 ; 178** |  |  |
| GR0721 | Rheinwald | ***** | **Caib-DRB*1 ; Caib-DRB*1** | **178 ; 178** | **A ; A** | ***** |
| GR0727 | Rheinwald |  | **Caib-DRB*1 ; Caib-DRB*1** | **174 ; 178** | **A ; A** |  |
| GR0728 | Rheinwald |  | **Caib-DRB*1** ; **Caib-DRB*2** | **174 ; 184** | **A ; G** | ***** |
| GR0729 | Rheinwald |  | **Caib-DRB*1 ; Caib-DRB*1** | **174 ; 174** | **A ; A** |  |
| GR0732 | Rheinwald | ***** | **Caib-DRB*1 ; Caib-DRB*1** | **178 ; 178** | **A ; A** | ***** |
| GR0766 | Rheinwald | ***** | **Caib-DRB*1 ; Caib-DRB*1** | **174 ; 178** | **A ; A** | ***** |
| GR0787 | Rheinwald | ***** | **Caib-DRB*1** ; **Caib-DRB*2** | **174 ; 184** | **A ; G** |  |
| GR0799 | Adula-Vial |  | **Caib-DRB*1 ; Caib-DRB*1** | **178 ; 178** |  |  |
| GR0806 | Albris |  | **Caib-DRB*1** ; **Caib-DRB*2** | **178 ; 184** | **A ; G** |  |
| GR0853 | Albris |  | **Caib-DRB*1** ; **Caib-DRB*2** | **174 ; 184** | **A ; G** |  |
| GR0863 | Val Bever |  | **Caib-DRB*1 ; Caib-DRB*1** | **174 ; 174** |  |  |
| GR0868 | Calanda |  | **Caib-DRB*2 ; Caib-DRB*2** | **184 ; 184** |  |  |
| GR0872 | Albris |  | **Caib-DRB*1 ; Caib-DRB*1** | **174 ; 178** |  |  |
| GR0875 | Albris |  | **Caib-DRB*1 ; Caib-DRB*1** | **174 ; 178** | **A ; A** |  |
| GR0953 | Rheinwald |  | **Caib-DRB*1** ; **Caib-DRB*2** | **174 ; 184** | **A ; G** |  |
| GR0987 | Rheinwald |  | **Caib-DRB*1** ; **Caib-DRB*2** | **174 ; 184** | **A ; G** |  |
| GR0989 | Rheinwald |  | **Caib-DRB*1 ; Caib-DRB*1** | **174 ; 178** | **A ; A** |  |
| GR1382 | Rheinwald |  | **Caib-DRB*1** ; **Caib-DRB*2** | **178 ; 184** | **A ; G** |  |
| GR1390 | Rheinwald |  | **Caib-DRB*1** ; **Caib-DRB*2** | **174 ; 184** | **A ; G** | ***** |
| GR1424 | Rheinwald |  | **Caib-DRB*1** ; **Caib-DRB*2** | **174 ; 184** | **A ; G** | ***** |
| GR1427 | Rheinwald |  | NaN | **174 ; 174** | **A ; A** |  |
| VD0001 | Cape au Moine |  | **Caib-DRB*1** ; **Caib-DRB*2** | NaN | **A ; G** |  |
| VD0004 | Cape au Moine |  | **Caib-DRB*1 ; Caib-DRB*1** | **174 ; 178** | **A ; A** |  |
| VD0005 | Cape au Moine |  | **Caib-DRB*1 ; Caib-DRB*1** | **174 ; 178** | **A ; A** |  |
| VD0007 | Cape au Moine |  | **Caib-DRB*1** ; **Caib-DRB*2** | **174 ; 184** | **A ; G** |  |
| VD0013 | Cape au Moine |  | **Caib-DRB*1** ; **Caib-DRB*2** | **174 ; 184** | **A ; G** |  |
| VD0023 | Cape au Moine |  | **Caib-DRB*1** ; **Caib-DRB*2** | **174 ; 184** | **A ; G** |  |
| VD0030 | Cape au Moine |  | **Caib-DRB*1 ; Caib-DRB*1** | **170 ; 174** | **A ; A** | ***** |
| VD0037 | Cape au Moine |  | **Caib-DRB*1 ; Caib-DRB*1** | **174 ; 174** | **A ; A** |  |
| VD0038 | Cape au Moine | ***** | **Caib-DRB*1** ; **Caib-DRB*2** | **178 ; 184** | **A ; G** | ***** |
| VD0039 | Cape au Moine | ***** | **Caib-DRB*1** ; **Caib-DRB*2** | **174 ; 184** | **A ; G** | ***** |
| VD0043 | Pierreuse-Gummfluh |  | **Caib-DRB*1 ; Caib-DRB*1** | **174 ; 178** |  |  |
| VD0044 | Pierreuse-Gummfluh |  | **Caib-DRB*1 ; Caib-DRB*1** | **174 ; 174** |  |  |
| VD0045 | Pierreuse-Gummfluh |  | **Caib-DRB*1 ; Caib-DRB*1** | **170 ; 178** |  |  |
| VD0048 | Cape au Moine |  | **Caib-DRB*1** ; **Caib-DRB*2** | **178 ; 184** | **A ; G** |  |
| VD0050 | Cape au Moine | ***** | **Caib-DRB*1** ; **Caib-DRB*2** | **174 ; 184** | **A ; G** |  |
| VD0052 | Cape au Moine | ***** | **Caib-DRB*1** ; **Caib-DRB*2** | **174 ; 184** | **A ; G** | ***** |
| VD0055 | Cape au Moine |  | **Caib-DRB*1 ; Caib-DRB*1** | **174 ; 178** | **A ; A** |  |
| VD0056 | Cape au Moine |  | **Caib-DRB*1 ; Caib-DRB*1** | **174 ; 174** | **A ; A** |  |
| VD0057 | Cape au Moine | ***** | **Caib-DRB*1** ; **Caib-DRB*2** | **174 ; 184** | **A ; G** | ***** |
| VD0058 | Cape au Moine | ***** | **Caib-DRB*1** ; **Caib-DRB*2** | **178 ; 184** | **A ; G** | ***** |
| VD0059 | Cape au Moine | ***** | **Caib-DRB*1** ; **Caib-DRB*2** | **178 ; 184** | **A ; G** |  |
| VD0060 | Cape au Moine | ***** | **Caib-DRB*1** ; **Caib-DRB*2** | **174 ; 184** | **A ; G** | ***** |
| VD0067 | Cape au Moine |  | **Caib-DRB*1** ; **Caib-DRB*2** | **174 ; 184** | **A ; G** | ***** |
| VD0129 | Cape au Moine |  | **Caib-DRB*1 ; Caib-DRB*1** | NaN | **A ; A** |  |
| VD0195 | Cape au Moine |  | **Caib-DRB*1 ; Caib-DRB*1** | **170 ; 174** | **A ; A** |  |
| VD0227 | Cape au Moine |  | **Caib-DRB*1 ; Caib-DRB*1** | **174 ; 174** | **A ; A** |  |
| VD0229 | Cape au Moine |  | **Caib-DRB*1 ; Caib-DRB*1** | NaN | **A ; A** |  |
| VS0006 | Weisshorn |  | **Caib-DRB*1 ; Caib-DRB*1** | **174 ; 174** | **A ; A** |  |
| VS0010 | Weissmies |  | **Caib-DRB*1 ; Caib-DRB*1** | **174 ; 174** |  |  |
| VS0021 | Gr.Lohner |  | **Caib-DRB*1 ; Caib-DRB*1** | **170 ; 170** |  |  |
| VS0031 | Weisshorn | ***** | **Caib-DRB*1** ; **Caib-DRB*2** | **174 ; 184** | **A ; G** | ***** |
| VS0034  VS0035 | Weisshorn  Weisshorn |  | **Caib-DRB*1 ; Caib-DRB*1**  **Caib-DRB*1 ; Caib-DRB*1** | **170 ; 174**  **170 ; 174** | **A ; A**  **A ; A** | ***** |
| VS0036 | Weisshorn |  | **Caib-DRB*1 ; Caib-DRB*1** | **174 ; 174** | **A ; A** |  |
| VS0037 | Weisshorn |  | **Caib-DRB*1 ; Caib-DRB*1** | **170 ; 174** | **A ; A** | ***** |
| VS0056 | Weisshorn |  | **Caib-DRB*1 ; Caib-DRB*1** | NaN | **A ; A** |  |
| VS0064 | Weisshorn |  | **Caib-DRB*1 ; Caib-DRB*1** | **174 ; 174** | **A ; A** |  |
| VS0079 | Weisshorn |  | **Caib-DRB*1 ; Caib-DRB*1** | **170 ; 174** | **A ; A** | ***** |
| VS0081 | Weisshorn |  | **Caib-DRB*1 ; Caib-DRB*1** | NaN | **A ; A** | ***** |
| VS0083 | Weisshorn |  | **Caib-DRB*1 ; Caib-DRB*1** | NaN | **A ; A** |  |
| VS0084 | Weisshorn |  | **Caib-DRB*1 ; Caib-DRB*1** | **174 ; 174** | **A ; A** |  |
| VS0102 | Mischabel |  | **Caib-DRB*1 ; Caib-DRB*1** | **170 ; 170** |  |  |
| VS0108 | Weissmies |  | **Caib-DRB*1 ; Caib-DRB*1** | NaN |  |  |
| VS0109 | Weissmies |  | **Caib-DRB*1 ; Caib-DRB*1** | **174 ; 174** |  |  |
| VS0110 | Weissmies |  | **Caib-DRB*1 ; Caib-DRB*1** | **174 ; 174** |  |  |
| VS0111 | Weissmies |  | **Caib-DRB*1 ; Caib-DRB*1** | **170 ; 174** |  |  |
| VS0112 | Weissmies |  | **Caib-DRB*1 ; Caib-DRB*1** | **170 ; 170** |  |  |
| VS0113 | Weissmies |  | **Caib-DRB*1 ; Caib-DRB*1** | **170 ; 170** |  |  |
| VS0114 | Weissmies |  | **Caib-DRB*1 ; Caib-DRB*1** | **174 ; 174** |  |  |
| VS0134 | Arolla |  | **Caib-DRB*1 ; Caib-DRB*1** | **170 ; 174** |  |  |
| VS0135 | Arolla |  | **Caib-DRB*1 ; Caib-DRB*1** | **170 ; 174** |  |  |
| VS0136 | Arolla |  | **Caib-DRB*1 ; Caib-DRB*1** | **170 ; 174** |  |  |
| VS0139 | Pleureur |  | **Caib-DRB*1 ; Caib-DRB*1** | **170 ; 170** |  |  |
| VS0163 | Pleureur |  | **Caib-DRB*1 ; Caib-DRB*1** | **170 ; 170** |  |  |
| VS0165 | Dents du Midi |  | **Caib-DRB*1 ; Caib-DRB*1** | **170 ; 174** |  |  |
| VS0166 | Tanay |  | **Caib-DRB*1 ; Caib-DRB*1** | **170 ; 174** |  |  |
| VS0167 | Pleureur |  | **Caib-DRB*1 ; Caib-DRB*1** | **170 ; 170** |  |  |
| VS0168 | Dents du Midi |  | **Caib-DRB*1 ; Caib-DRB*1** | **170 ; 170** |  |  |
| VS0175 | Pleureur | ***** | **Caib-DRB*1 ; Caib-DRB*1** | **170 ; 174** |  |  |
| VS0179 | Pleureur |  | **Caib-DRB*1 ; Caib-DRB*1** | **174 ; 174** |  |  |
| VS0238 | Arolla |  | **Caib-DRB*1 ; Caib-DRB*1** | **170 ; 174** |  |  |
| VS0246 | Pleureur |  | **Caib-DRB*1 ; Caib-DRB*1** | **170 ; 174** |  |  |
| VS0249 | Arolla |  | **Caib-DRB*1 ; Caib-DRB*1** | **174 ; 174** |  |  |
| VS0254 | Tanay |  | **Caib-DRB*1 ; Caib-DRB*1** | **170 ; 174** |  |  |
| VS0258 | Dents du Midi |  | **Caib-DRB*1 ; Caib-DRB*1** | **174 ; 174** |  |  |
| VS0271 | Tanay |  | **Caib-DRB*1 ; Caib-DRB*1** | **170 ; 170** |  |  |
| VS0295 | Weisshorn |  | **Caib-DRB*1 ; Caib-DRB*1** | NaN | **A ; A** |  |
| VS0326 | Muveran |  | **Caib-DRB*1 ; Caib-DRB*1** | **174 ; 178** |  |  |
| VS0337 | Rawyl |  | **Caib-DRB*1 ; Caib-DRB*1** | **170 ; 178** |  |  |
| VS0362 | Diablerets |  | **Caib-DRB*1 ; Caib-DRB*1** | **174 ; 178** |  |  |
| VS0374 | Rawyl |  | **Caib-DRB*1 ; Caib-DRB*1** | **170 ; 178** |  |  |
| VS0381 | Rawyl |  | **Caib-DRB*1 ; Caib-DRB*1** | **174 ; 174** |  |  |
| VS0434 | Aletsch-Bietschhorn |  | **Caib-DRB*1 ; Caib-DRB*1** | NaN |  |  |
| VS0435 | Aletsch-Bietschhorn |  | **Caib-DRB*1 ; Caib-DRB*1** | **170 ; 170** |  |  |
| VS0440 | Aletsch-Bietschhorn |  | **Caib-DRB*1 ; Caib-DRB*1** | **174 ; 174** |  |  |
| VS0464 | Aletsch-Bietschhorn |  | **Caib-DRB*1 ; Caib-DRB*1** | **170 ; 174** |  |  |
| VS0465 | Aletsch-Bietschhorn |  | **Caib-DRB*1 ; Caib-DRB*1** | **174 ; 174** |  |  |
| VS0466 | Aletsch-Bietschhorn |  | **Caib-DRB*1 ; Caib-DRB*1** | **170 ; 174** |  |  |
| VS0467 | Aletsch-Bietschhorn |  | **Caib-DRB*1 ; Caib-DRB*1** | **170 ; 170** |  |  |
| VS0488 | Weisshorn |  | **Caib-DRB*1 ; Caib-DRB*1** | NaN | **A ; A** |  |
| VS0491 | Weisshorn |  | **Caib-DRB*1 ; Caib-DRB*1** | NaN | **A ; A** | ***** |
| VS0492 | Weisshorn |  | **Caib-DRB*1 ; Caib-DRB*1** | NaN | **A ; A** | ***** |
| VS0515 | Weisshorn |  | **Caib-DRB*1 ; Caib-DRB*1** | NaN | **A ; A** | ***** |
| VS0518 | Weisshorn |  | **Caib-DRB*1 ; Caib-DRB*1** | NaN | **A ; A** |  |
| VS0525 | Weisshorn |  | **Caib-DRB*1 ; Caib-DRB*1** | NaN |  |  |
| VS0551 | Weisshorn |  | **Caib-DRB*1 ; Caib-DRB*1** | NaN | **A ; A** |  |
| VS0652 | Weisshorn |  | **Caib-DRB*1 ; Caib-DRB*1** | NaN | **A ; A** |  |
| VS0666 | Weisshorn |  | **Caib-DRB*1 ; Caib-DRB*1** | NaN | **A ; A** |  |
| VS0670 | Weisshorn |  | **Caib-DRB*1 ; Caib-DRB*1** | NaN | **A ; A** |  |
| VS0672 | Weisshorn |  | **Caib-DRB*1 ; Caib-DRB*1** | NaN | **A ; A** |  |
