# Supplementary material for: Introgression from Domestic Goat Generated Variation at the Major Histocompatibility Complex of Alpine Ibex
Source: PLoS Genet. 2014 Jun 19;10(6):e1004438. doi: 10.1371/journal.pgen.1004438 (PMC4063738; doi:10.1371/journal.pgen.1004438)
Supplement: Table S5 — Sample sizes of Alpine ibex populations and domestic goat breeds included in the SNP genotyping. The allele frequencies at SNP16397 (diagnostic for Caib-DRB*2) are shown for each population and breed, respectively. (DOCX) [file pgen.1004438.s012.docx]

**Table S5:** Sample sizes of Alpine ibex populations and domestic goat breeds included in the SNP genotyping. The allele frequencies at SNP16397 (diagnostic for *Caib-DRB*2*) are shown for each population and breed, respectively.

| **Alpine ibex populations** | | *n individuals*  *SNP genotyping* | *n individuals*  *genotyped at SNP16397* | | *Frequency of SNP16397/G* |
| --- | --- | --- | --- | --- | --- |
| Albris | | 24 | 21 | | 0.25 |
| Cape au Moine | | 24 | 24 | | 0.30 |
| Rheinwald | | 24 | 24 | | 0.30 |
| Weisshorn | | 23 | 23 | | 0.02 |
|  |  | |  | |  |
| **Domestic goat breeds** | | |  | |  |
| Buendner Strahlenziege | | 20 | 20 | | 0.58 |
| Capra Grigia | | 20 | 20 | | 0.63 |
| Gemsfarbige Gebirgsziege | | 32 | 32 | | 0.34 |
| Kupferziege | | 36 | 25 | | 0.39 |
| Nera Verzasga Ziege | | 20 | 20 | | 0.68 |
| Saanenziege | | 49 | 48 | | 0.30 |
|  | |  |  | |  |
| Overall | | 272 | 267 |  | |
