# Supplementary material for: Introgression from Domestic Goat Generated Variation at the Major Histocompatibility Complex of Alpine Ibex
Source: PLoS Genet. 2014 Jun 19;10(6):e1004438. doi: 10.1371/journal.pgen.1004438 (PMC4063738; doi:10.1371/journal.pgen.1004438)
Supplement: Table S6 — Microsatellites linked to the MHC DRB locus. Names in italic and brackets are synonyms for the microsatellite names used in the text. 1) Chromosomal location and gene region; 2) Ta: annealing temperature; 3) Number of cycles. (DOCX) [file pgen.1004438.s013.docx]

**Table S6:** Microsatellites linked to the MHC *DRB* locus. Names in italic and brackets are synonyms for the microsatellite names used in the text. ^1)^ Chromosomal location and gene region; ^2)^ T_a_: annealing temperature; ^3)^ Number of cycles.

| Microsatellite | Location^1^ | T_a_ [°C]^2^ (cycles)^3^ | Primer sequence (5’-3’) | References |
| --- | --- | --- | --- | --- |
| OLADRB1 *(DRB1)* | chr23, MHC II | 62 (35) | F: TGT GCA GCG GCG AGG TGA G R: CGT ACC CAG AGA KTG AGT GAA GTA TC | [1,2] |
| OLADRB2 (OLADRBp,  OLADRB) | chr23, MHC II | 60 (35) | F: CTG CCA ATG CAG AGA CAC AAG A R: GTC TGT CTC CTG TCT TGT CAT C | [3], Genbank: UniSTS:251420 |

1. Schwaiger F, Buitkamp J, Weyers E, Epplen J (1993) Typing of Artiodactyl MHC-*DRB* genes with the help of intronic simple repeated DNA-sequences. Mol Ecol 2: 55–59.

2. Paterson S, Wilson K, Pemberton JM (1998) Major histocompatibility complex variation associated with juvenile survival and parasite resistance in a large unmanaged ungulate population. Proc Natl Acad Sci U S A 95: 3714–3719.

3. Blattman A, Beh K (1992) Dinucleotide repeat polymorphism within the ovine major histocompatibility complex. Anim Genet 23: 392–392.
