# Supplementary material for: Introgression from Domestic Goat Generated Variation at the Major Histocompatibility Complex of Alpine Ibex
Source: PLoS Genet. 2014 Jun 19;10(6):e1004438. doi: 10.1371/journal.pgen.1004438 (PMC4063738; doi:10.1371/journal.pgen.1004438)
Supplement: Table S7 — Primer sequences used for amplifying four loci of the MHC DRB gene of Alpine ibex and domestic goat. Locus “Intron 4 - Exon 6” corresponds to the locus spanning partial intron 4, exon 5, intron 5, exon 6, 3′ UTR and was amplified using two primer pairs. (DOCX) [file pgen.1004438.s014.docx]

**Table S7:** Primer sequences used for amplifying four loci of the MHC *DRB* gene of Alpine ibex and domestic goat. Locus "Intron 4 - Exon 6" corresponds to the locus spanning partial intron 4, exon 5, intron 5, exon 6, 3' UTR and was amplified using two primer pairs.

| **Primer Name** | **Primer Sequence (5’ – 3’)** | | **Locus** | **MHC *DRB* region amplified** |
| --- | --- | --- | --- | --- |
| DRB_intron1_F | GAGGTGAACATGTTTAAGGAACTG | Intron 1 | | partial intron 1 |
| DRB_intron1_R | CAGGAAATGTGCTGCAGAGA | Intron 1 | | partial intron 1 |
| DRB_intron2_F | TGCATTTTGTTTGTTCTTCTTTCTCT | Intron 2 | | partial intron 2 |
| DRB_intron2_R | TCCTGGGAAAGCTCTCTCAA | Intron 2 | | partial intron 2 |
| DRB_exon3_F | TCTGGCTTCAGAAATTATGCTTTTAAT | Exon 3 | | partial intron 2, exon 3, partial intron 3 |
| DRB_exon3_R | ATCACGGGAAGCTCAGAACA | Exon 3 | | partial intron 2, exon 3, partial intron 3 |
| DRB_intron4_F | GCTGACTTAATTGCATTGGATTGG | Intron 4 - Exon 6 | | partial intron 4, exon 5, partial intron 5 |
| DRB_intron5_R | CCAGACATAACTCTACAGGGGA | Intron 4 - Exon 6 | | partial intron 4, exon 5, partial intron 5 |
| DRB_exon6_F | CCCCTGTAGAGTTATGTCTGGA | Intron 4 - Exon 6 | | partial intron 5, exon 6, 3' UTR |
| DRB_exon6_R | TGAGCTCAGATGCACAGGAA | Intron 4 - Exon 6 | | partial intron 5, exon 6, 3' UTR |
