# Supplementary material for: Introgression from Domestic Goat Generated Variation at the Major Histocompatibility Complex of Alpine Ibex
Source: PLoS Genet. 2014 Jun 19;10(6):e1004438. doi: 10.1371/journal.pgen.1004438 (PMC4063738; doi:10.1371/journal.pgen.1004438)
Supplement: Text S1 — MHC DRB sequence alignment. Partial intron 1. (DOC) [file pgen.1004438.s015.doc]

**MHC DRB sequence alignment**

partial intron 1

++ intron 1 ++++++++++++++++++++++++++++++++++++++++++++++++

DRB*1_GR0150 ttaaggaactgttaaaagagctgcaggatgaagggagagcactggttttccggtggagga

DRB*1_GR0701 ttaaggaactgttaaaagagctgcaggatgaagggagagcactggttttccggtggagga

DRB*1_GR0721 ttaaggaactgttaaaagagctgcaggatgaagggagagcactggttttccggtggagga

DRB*1_GR0732 ttaaggaactgttaaaagagctgcaggatgaagggagagcactggttttccggtggagga

DRB*1_VD0030 ttaaggaactgttaaaagagctgcaggatgaagggagagcactggttttccggtggagga

DRB*1_VS0112 ttaaggaactgttaaaagagctgcaggatgaagggagagcactggttttccggtggagga

DRB*1_VS0139 ttaaggaactgttaaaagagctgcaggatgaagggagagcactggttttccggtggagga

DRB*2_GR0023 ttaaggaactgttaaaagagctgcaggatgacaggagagcgctggttttctggtggagga

DRB*2_GR0034 ttaaggaactgttaaaagagctgcaggatgacaggagagcgctggttttctggtggagga

DRB*2_GR0065 ttaaggaactgttaaaagagctgcaggatgacaggagagcgctggttttctggtggagga

DRB*2_GR0140 ttaaggaactgttaaaagagctgcaggatgacaggagagcgctggttttctggtggagga

DRB*2_GR0201 ttaaggaactgttaaaagagctgcaggatgacaggagagcgctggttttctggtggagga

DRB*2_GR0310 ttaaggaactgttaaaagagctgcaggatgacaggagagcgctggttttctggtggagga

DRB*2_GR0616 ttaaggaactgttaaaagagctgcaggatgacaggagagcgctggttttctggtggagga

goat_VBN4 ttaaggaactgttaaaagagctgcaggatgacaggagagcgctggttttctggtggagga

goat_ALP1.F01 ttaaggaactgttaaaagagctgcaggatgacgggagagcactggttttctggtggagga

goat_ALP1.E02 nnnnnnnnnnnnnnnnnnnnnnnnnnnnnnnnnnnnnnnnnctggttttctagtggagga

goat_ALP1.C02 ttaaggaactgttgaaagagctgcaggatgacgcgagcgcgctggttttctagtggagga

goat_GRS.A04 ttaaggaactgttgaaagagctgnaggatgacgsgagagcgctggttttctrgtggagga

++ intron 1 ++++++++++++++++++++++++++++++++++++++++++++++++

DRB*1_GR0150 ttctaggaagaggt-----------------------------------------tcagg

DRB*1_GR0701 ttctaggaagaggt-----------------------------------------tcagg

DRB*1_GR0721 ttctaggaagaggt-----------------------------------------tcagg

DRB*1_GR0732 ttctaggaagaggt-----------------------------------------tcagg

DRB*1_VD0030 ttctaggaagaggt-----------------------------------------tcagg

DRB*1_VS0112 ttctaggaagaggt-----------------------------------------tcagg

DRB*1_VS0139 ttctaggaagaggt-----------------------------------------tcagg

DRB*2_GR0023 tt-taggaagaggtggtgaagaggcaaatcctctaagtcctcaggcttagaggactcagg

DRB*2_GR0034 tt-taggaagaggtggtgaagaggcaaatcctctaagtcctcaggcttagaggactcagg

DRB*2_GR0065 tt-taggaagaggtggtgaagaggcaaatcctctaagtcctcaggcttagaggactcagg

DRB*2_GR0140 tt-taggaagaggtggtgaagaggcaaatcctctaagtcctcaggcttagaggactcagg

DRB*2_GR0201 tt-taggaagaggtggtgaagaggcaaatcctctaagtcctcaggcttagaggactcagg

DRB*2_GR0310 tt-taggaagaggtggtgaagaggcaaatcctctaagtcctcaggcttagaggactcagg

DRB*2_GR0616 tt-taggaagaggtggtgaagaggcaaatcctctaagtcctcaggcttagaggactcagg

goat_VBN4 tt-taggaagaggtggtgaagaggcaaatcctctaagtcctcaggcttagaggactcagg

goat_ALP1.F01 ttctaggaagaggtggtgaaggggcaaatcctctaagtcctcaggcttagaggactcagg

goat_ALP1.E02 ttctaggaagaggtggtgaaggggcaaatccnntaagtcctcaggcttagaggactcagg

goat_ALP1.C02 ttctaggaagaggtggtgaaggggcaaatcctctaagtcctcaggcttagaggactcagg

goat_GRS.A04 tt-taggaagaggtggtgaagrggcaaatcctctaagtcctcaggcttagaggactcagg

++ intron 1 ++++++++++++++++++++++++++++++++++++++++++++++++

DRB*1_GR0150 cctagagggctccagccaagcatggaagctggactaggtcgggaaaggcttggataagag

DRB*1_GR0701 cctagagggctccagccaagcatggaagctggactaggtcgggaaaggcttggataagag

DRB*1_GR0721 cctagagggctccagccaagcatggaagctggactaggtcgggaaaggcttggataagag

DRB*1_GR0732 cctagagggctccagccaagcatggaagctggactaggtcgggaaaggcttggataagag

DRB*1_VD0030 cctagagggctccagccaagcatggaagctggactaggtcgggaaaggcttggataagag

DRB*1_VS0112 cctagagggctccagccaagcatggaagctggactaggtcgggaaaggcttggataagag

DRB*1_VS0139 cctagagggctccagccaagcatggaagctggactaggtcgggaaaggcttggataagag

DRB*2_GR0023 cctagagggctccagccaagcatggaagctggactaggtcgggaaaggcttggataggag

DRB*2_GR0034 cctagagggctccagccaagcatggaagctggactaggtcgggaaaggcttggataggag

DRB*2_GR0065 cctagagggctccagccaagcatggaagctggactaggtcgggaaaggcttggataggag

DRB*2_GR0140 cctagagggctccagccaagcatggaagctggactaggtcgggaaaggcttggataggag

DRB*2_GR0201 cctagagggctccagccaagcatggaagctggactaggtcgggaaaggcttggataggag

DRB*2_GR0310 cctagagggctccagccaagcatggaagctggactaggtcgggaaaggcttggataggag

DRB*2_GR0616 cctagagggctccagccaagcatggaagctggactaggtcgggaaaggcttggataggag

goat_VBN4 cctagagggctccagccaagcatggaagctggactaggtcgggaaaggcttggataggag

goat_ALP1.F01 cctagagggctccagcc-agcatggaagctggactaggtggggaaaggcttggataggag

goat_ALP1.E02 cctagagggctccagcc-agcatggaagctggactaggtsgggaaaggcttggataggag

goat_ALP1.C02 cctagagggctccagcc-agcatggaagctggactaggtcgggaaaggcttggataggac

goat_GRS.A04 cctagagggctctaggcaagcatggaagctggactaggtcgggaaaggcttggataagag

++ intron 1 ++++++++++++++++++++++++++++++++++++++++++++++++

DRB*1_GR0150 tgatttctcttggcctcccacagcctcagtctgttgagcgaagatgaaagaactcaaagc

DRB*1_GR0701 tgatttctcttggcctcccacagcctcagtctgttgagcgaagatgaaagaactcaaagc

DRB*1_GR0721 tgatttctcttggcctcccacagcctcagtctgttgagcgaagatgaaagaactcaaagc

DRB*1_GR0732 tgatttctcttggcctcccacagcctcagtctgttgagcgaagatgaaagaactcaaagc

DRB*1_VD0030 tgatttctcttggcctcccacagcctcagtctgttgagcgaagatgaaagaactcaaagc

DRB*1_VS0112 tgatttctcttggcctcccacagcctcagtctgttgagcgaagatgaaagaactcaaagc

DRB*1_VS0139 tgatttctcttggcctcccacagcctcagtctgttgagcgaagatgaaagaactcaaagc

DRB*2_GR0023 agatttccccaagcctcccgcagccttcctctgttgagagaagatgaaagaactcaaggc

DRB*2_GR0034 agatttccccaagcctcccgcagccttcctctgttgagagaagatgaaagaactcaaggc

DRB*2_GR0065 agatttccccaagcctcccgcagccttcctctgttgagagaagatgaaagaactcaaggc

DRB*2_GR0140 agatttccccaagcctcccgcagccttcctctgttgagagaagatgaaagaactcaaggc

DRB*2_GR0201 agatttccccaagcctcccgcagccttcctctgttgagagaagatgaaagaactcaaggc

DRB*2_GR0310 agatttccccaagcctcccgcagccttcctctgttgagagaagatgaaagaactcaaggc

DRB*2_GR0616 agatttccccaagcctcccgcagccttcctctgttgagagaagatgaaagaactcaaggc

goat_VBN4 agatttccccaagcctcccgcagccttcctctgttgagagaagatgaaagaactcaaggc

goat_ALP1.F01 agatttccccaggcctcccgtagcctccgtctgttgagcgaagatggaagaacctgaggt

goat_ALP1.E02 agatttccccaggcctcccgyagcctccgtctgttgagcgaagatggaagaacctgaggt

goat_ALP1.C02 agatttccccaggcctcccgcagcctccgtctgttgagcgaagatggaagaacctgaggt

goat_GRS.A04 agatttcccctggcctcccgcagcctcagtctgttgagtgaagatgaaagaactcaaagc

++ intron 1 ++++++++++++++++++++++++++++++++++++++++++++++++

DRB*1_GR0150 gtgctggtagttggggtgccggtggagccagcgaggggcttagggctagtcttggcggct

DRB*1_GR0701 gtgctggtagttggggtgccggtggagccagcgaggggcttagggctagtcttggcggct

DRB*1_GR0721 gtgctggtagttggggtgccggtggagccagcgaggggcttagggctagtcttggcggct

DRB*1_GR0732 gtgctggtagttggggtgccggtggagccagcgaggggcttagggctagtcttggcggct

DRB*1_VD0030 gtgctggtagttggggtgccggtggagccagcgaggggcttagggctagtcttggcggct

DRB*1_VS0112 gtgctggtagttggggtgccggtggagccagcgaggggcttagggctagtcttggcggct

DRB*1_VS0139 gtgctggtagttggggtgccggtggagccagcgaggggcttagggctagtcttggcggct

DRB*2_GR0023 gtgctggtagttggggtgcc-gtggagccagcgagggggttagggctagtcctagcggcc

DRB*2_GR0034 gtgctggtagttggggtgcc-gtggagccagcgagggggttagggctagtcctagcggcc

DRB*2_GR0065 gtgctggtagttggggtgcc-gtggagccagcgagggggttagggctagtcctagcggcc

DRB*2_GR0140 gtgctggtagttggggtgcc-gtggagccagcgagggggttagggctagtcctagcggcc

DRB*2_GR0201 gtgctggtagttggggtgcc-gtggagccagcgagggggttagggctagtcctagcggcc

DRB*2_GR0310 gtgctggtagttggggtgcc-gtggagccagcgagggggttagggctagtcctagcggcc

DRB*2_GR0616 gtgctggtagttggggtgcc-gtggagccagcgagggggttagggctagtcctagcggcc

goat_VBN4 gtgctggtagttggggtgcc-gtggagccagcgagggggttagggctagtcctagcggcc

goat_ALP1.F01 gtgctggtagttggggtgccagtggagccagcgagggggttagggctagtcctagtggcc

goat_ALP1.E02 gtgctggtagttggggtgccagtggagccagcgagggggttagggctagtcctagtggcc

goat_ALP1.C02 gtgctggtagttggggtgccagtggagccagcgagggggttagggctagtcctagtggcc

goat_GRS.A04 gttctggtagttggggtgccggtggagccagagagggggttagggctaatcctagcggcc

++ intron 1 ++++++++++++++++++++++++++++++++++++++++++++++++

DRB*1_GR0150 ccagcagagcctgggcgtcccacattggtgggtgtcgctcctgccctccatccctc---a

DRB*1_GR0701 ccagcagagcctgggcgtcccacattggtgggtgtcgctcctgccctccatccctc---a

DRB*1_GR0721 ccagcagagcctgggcgtcccacattggtgggtgtcgctcctgccctccatccctc---a

DRB*1_GR0732 ccagcagagcctgggcgtcccacattggtgggtgtcgctcctgccctccatccctc---a

DRB*1_VD0030 ccagcagagcctgggcgtcccacattggtgggtgtcgctcctgccctccatccctc---a

DRB*1_VS0112 ccagcagagcctgggcgtcccacattggtgggtgtcgctcctgccctccatccctc---a

DRB*1_VS0139 ccagcagagcctgggcgtcccacattggtgggtgtcgctcctgccctccatccctc---a

DRB*2_GR0023 ccaactgagcctgggcgtccggcattggtgggtgtcacccctgccctccatccgttatta

DRB*2_GR0034 ccaactgagcctgggcgtccggcattggtgggtgtcacccctgccctccatccgttatta

DRB*2_GR0065 ccaactgagcctgggcgtccggcattggtgggtgtcacccctgccctccatccgttatta

DRB*2_GR0140 ccaactgagcctgggcgtccggcattggtgggtgtcacccctgccctccatccgttatta

DRB*2_GR0201 ccaactgagcctgggcgtccggcattggtgggtgtcacccctgccctccatccgttatta

DRB*2_GR0310 ccaactgagcctgggcgtccggcattggtgggtgtcacccctgccctccatccgttatta

DRB*2_GR0616 ccaactgagcctgggcgtccggcattggtgggtgtcacccctgccctccatccgttatta

goat_VBN4 ccaactgagcctgggcgtccggcattggtgggtgtcacccctgccctccatccgttatta

goat_ALP1.F01 ctaactgagcctgggcgtcccgcatt-atgggtgtcgcccctgcccgccatccctcattg

goat_ALP1.E02 cyaactgagcctgggcgtcccgcatt-atgggtgtcgcccctgcccgccatccctcattg

goat_ALP1.C02 ccaactgagcctgggcgtcccgcatt-atgggtgtcgcccctgcccgccatccctcattg

goat_GRS.A04 ccaactgagcctggncntcccgcattggtgggtgtcgcccctgccctccatccctyttta

++ intron 1 +++++++++++++++++++++++++++++++++

DRB*1_GR0150 gcctctctccaggagtccgctcctgtgaccagatctatcctctct

DRB*1_GR0701 gcctctctccaggagtccgctcctgtgaccagatctatcctctct

DRB*1_GR0721 gcctctctccaggagtccgctcctgtgaccagatctatcctctct

DRB*1_GR0732 gcctctctccaggagtccgctcctgtgaccagatctatcctctct

DRB*1_VD0030 gcctctctccaggagtccgctcctgtgaccagatctatcctctct

DRB*1_VS0112 gcctctctccaggagtccgctcctgtgaccagatctatcctctct

DRB*1_VS0139 gcctctctccaggagtccgctcctgtgaccagatctatcctctct

DRB*2_GR0023 gcctccccccaggagtccgctcctgtgaccagatctatcccgtct

DRB*2_GR0034 gcctccccccaggagtccgctcctgtgaccagatctatcccgtct

DRB*2_GR0065 gcctccccccaggagtccgctcctgtgaccagatctatcccgtct

DRB*2_GR0140 gcctccccccaggagtccgctcctgtgaccagatctatcccgtct

DRB*2_GR0201 gcctccccccaggagtccgctcctgtgaccagatctatcccgtct

DRB*2_GR0310 gcctccccccaggagtccgctcctgtgaccagatctatcccgtct

DRB*2_GR0616 gcctccccccaggagtccgctcctgtgaccagatctatcccgtct

goat_VBN4 gcctccccccaggagtccgctcctgtgaccagatctatcccgtct

goat_ALP1.F01 gcctctccccaggagtccgctcctgtgaccagatctatcctctct

goat_ALP1.E02 gcctctccccaggagtccgctcctgtgaccagatctatcctstct

goat_ALP1.C02 gcctctccccaggagtccgctcctgtgaccagatctatcccgtct

goat_GRS.A04 gcctccccccaggagtccgctcctgtgaccagatctatcctctct
