# Supplementary material for: Introgression from Domestic Goat Generated Variation at the Major Histocompatibility Complex of Alpine Ibex
Source: PLoS Genet. 2014 Jun 19;10(6):e1004438. doi: 10.1371/journal.pgen.1004438 (PMC4063738; doi:10.1371/journal.pgen.1004438)
Supplement: Text S2 — MHC DRB sequence alignment. Partial intron 2. (DOC) [file pgen.1004438.s016.doc]

**MHC DRB sequence alignment**

partial intron 2

++ intron 2 ++++++++++++++++++++++++++++++++++++++++++++++++

DRB*1_GR0150 acaggctgtggattgttgcatttgccttgtcatttgtttctaggtattttttt-atttct

DRB*1_GR0701 acaggctgtggattgttgcatttgccttgtcatttgtttctaggtattttttt-atttct

DRB*1_GR0721 acaggctgtggattgttgcatttgccttgtcatttgtttctaggtattttttt-atttct

DRB*1_GR0732 acaggctgtggattgttgcatttgccttgtcatttgtttctaggtattttttt-atttct

DRB*1_VD0030 acaggctgtggattgttgcatttgccttgtcatttgtttctaggtattttttt-atttct

DRB*1_VS0112 acaggctgtggattgttgcatttgccttgtcatttgtttctaggtattttttt-atttct

DRB*1_VS0139 acaggctgtggattgttgcatttgccttgtcatttgtttctaggtattttttt-atttct

DRB*2_GR0023 acaggctgtgggttgttgcatttgccttgtcatttgtttctaggtatttttttaatttct

DRB*2_GR0034 acaggctgtgggttgttgcatttgccttgtcatttgtttctaggtatttttttaatttct

DRB*2_GR0065 acaggctgtgggttgttgcatttgccttgtcatttgtttctaggtatttttttaatttct

DRB*2_GR0140 acaggctgtgggttgttgcatttgccttgtcatttgtttctaggtatttttttaatttct

DRB*2_GR0201 acaggctgtgggttgttgcatttgccttgtcatttgtttctaggtatttttttaatttct

DRB*2_GR0310 acaggctgtgggttgttgcatttgccttgtcatttgtttctaggtatttttttaatttct

DRB*2_GR0616 acaggctgtgggttgttgcatttgccttgtcatttgtttctaggtatttttttaatttct

goat_VBN4 acaggctgtgggttgttgcatttgccttgtcatttgtttctaggtatttttttaatttct

goat_ALP1.F01 acaggctgtggattgttgcatttgccttgtcatttgtttctaggtattttttt-atttct

goat_ALP1.E02 acaggctgtggattgttgcatttgccttgtcatttgtttctaggtattttttt-atttct

goat_ALP1.C02 acaggctgtggattgttgcatttgccctgtcgtttgtttcta-----tttttt-atttct

goat_GRS.A04 acaggctgtggattattgcatttgctttgtc--ttgtttctaggtatttttta-atttct

++ intron 2 ++++++++++++++++++++++++++++++++++++++++++++++++

DRB*1_GR0150 tctttaatttcttcattaatccattaattattcagtaacatactgtttagtctccatgtg

DRB*1_GR0701 tctttaatttcttcattaatccattaattattcagtaacatactgtttagtctccatgtg

DRB*1_GR0721 tctttaatttcttcattaatccattaattattcagtaacatactgtttagtctccatgtg

DRB*1_GR0732 tctttaatttcttcattaatccattaattattcagtaacatactgtttagtctccatgtg

DRB*1_VD0030 tctttaatttcttcattaatccattaattattcagtaacatactgtttagtctccatgtg

DRB*1_VS0112 tctttaatttcttcattaatccattaattattcagtaacatactgtttagtctccatgtg

DRB*1_VS0139 tctttaatttcttcattaatccattaattattcagtaacatactgtttagtctccatgtg

DRB*2_GR0023 tctttaatttcttcattaatccattgattattcagaaacatactgtttagcctccatgtg

DRB*2_GR0034 tctttaatttcttcattaatccattgattattcagaaacatactgtttagcctccatgtg

DRB*2_GR0065 tctttaatttcttcattaatccattgattattcagaaacatactgtttagcctccatgtg

DRB*2_GR0140 tctttaatttcttcattaatccattgattattcagaaacatactgtttagcctccatgtg

DRB*2_GR0201 tctttaatttcttcattaatccattgattattcagaaacatactgtttagcctccatgtg

DRB*2_GR0310 tctttaatttcttcattaatccattgattattcagaaacatactgtttagcctccatgtg

DRB*2_GR0616 tctttaatttcttcattaatccattgattattcagaaacatactgtttagcctccatgtg

goat_VBN4 tctttaatttcttcattaatccattgattattcagaaacatactgtttagcctccatgtg

goat_ALP1.F01 tctttaatttcttcattaattcattgattattcagtaacatactgtatagcctccatgtg

goat_ALP1.E02 tctttaatttcttcattaattcattgattattcagtaacatactgtatagcctccatgtg

goat_ALP1.C02 tttttaatttcttcattaatccattgattattcagtaacatactgtttagcctccatgtg

goat_GRS.A04 tctttaatttcttcattaatccattgcttattcagtaacatactgtttagcctccatgtg

++ intron 2 ++++++++++++++++++++++++++++++++++++++++++++++++

DRB*1_GR0150 tttgtgttttttacagtt-tttttttcctgtaattgatttctaatctcacagtgttgtgg

DRB*1_GR0701 tttgtgttttttacagtt-tttttttcctgtaattgatttctaatctcacagtgttgtgg

DRB*1_GR0721 tttgtgttttttacagtt-tttttttcctgtaattgatttctaatctcacagtgttgtgg

DRB*1_GR0732 tttgtgttttttacagtt-tttttttcctgtaattgatttctaatctcacagtgttgtgg

DRB*1_VD0030 tttgtgttttttacagtt-tttttttcctgtaattgatttctaatctcacagtgttgtgg

DRB*1_VS0112 tttgtgttttttacagtt-tttttttcctgtaattgatttctaatctcacagtgttgtgg

DRB*1_VS0139 tttgtgttttttacagtt-tttttttcctgtaattgatttctaatctcacagtgttgtgg

DRB*2_GR0023 tttttgttttttacagtt-tttttcccccataattgatttctaatctcacagtgttgtgg

DRB*2_GR0034 tttttgttttttacagtt-tttttcccccataattgatttctaatctcacagtgttgtgg

DRB*2_GR0065 tttttgttttttacagtt-tttttcccccataattgatttctaatctcacagtgttgtgg

DRB*2_GR0140 tttttgttttttacagtt-tttttcccccataattgatttctaatctcacagtgttgtgg

DRB*2_GR0201 tttttgttttttacagtt-tttttcccccataattgatttctaatctcacagtgttgtgg

DRB*2_GR0310 tttttgttttttacagtt-tttttcccccataattgatttctaatctcacagtgttgtgg

DRB*2_GR0616 tttttgttttttacagtt-tttttcccccataattgatttctaatctcacagtgttgtgg

goat_VBN4 tttttgttttttacagtt-tttttcccccataattgatttctaatctcacagtgttgtgg

goat_ALP1.F01 tttgtgttttttacagtt-tttttttcctgtaattgatttctaatctcacagcgttgtgg

goat_ALP1.E02 tttgtgttttttacagtt-tttttttcctgtaattgatttctaatctcacagcgttgtgg

goat_ALP1.C02 tttgtgtttttta---gt-ttttttccctgtaattgatttctaatctcacagtactgtgg

goat_GRS.A04 tttgtgttttttacagttattttttccctgtaatttatttctaatctcacagtgttgtgg

++ intron 2 ++++++++++++++++++++++++++++++++++++++++++++++++

DRB*1_GR0150 ttggaaaagatgctttagattaatcttaaatctttcatcttatgatattgtaaaaatgtc

DRB*1_GR0701 ttggaaaagatgctttagattaatcttaaatctttcatcttatgatattgtaaaaatgtc

DRB*1_GR0721 ttggaaaagatgctttagattaatcttaaatctttcatcttatgatattgtaaaaatgtc

DRB*1_GR0732 ttggaaaagatgctttagattaatcttaaatctttcatcttatgatattgtaaaaatgtc

DRB*1_VD0030 ttggaaaagatgctttagattaatcttaaatctttcatcttatgatattgtaaaaatgtc

DRB*1_VS0112 ttggaaaagatgctttagattaatcttaaatctttcatcttatgatattgtaaaaatgtc

DRB*1_VS0139 ttggaaaagatgctttagattaatcttaaatctttcatcttatgatattgtaaaaatgtc

DRB*2_GR0023 ttggaaaagatgctttagattaatcttaaatctttcatcttatgatattgtaaaaatgtc

DRB*2_GR0034 ttggaaaagatgctttagattaatcttaaatctttcatcttatgatattgtaaaaatgtc

DRB*2_GR0065 ttggaaaagatgctttagattaatcttaaatctttcatcttatgatattgtaaaaatgtc

DRB*2_GR0140 ttggaaaagatgctttagattaatcttaaatctttcatcttatgatattgtaaaaatgtc

DRB*2_GR0201 ttggaaaagatgctttagattaatcttaaatctttcatcttatgatattgtaaaaatgtc

DRB*2_GR0310 ttggaaaagatgctttagattaatcttaaatctttcatcttatgatattgtaaaaatgtc

DRB*2_GR0616 ttggaaaagatgctttagattaatcttaaatctttcatcttatgatattgtaaaaatgtc

goat_VBN4 ttggaaaagatgctttagattaatcttaaatctttcatcttatgatattgtaaaaatgtc

goat_ALP1.F01 ttggaaaagatgctttagattaatctgaaatctttcatcttatgatattgtaaaaa--tc

goat_ALP1.E02 ttggaaaagatgctttagattaatctgaaatctttcatcttatgatattgtaaaaa--tc

goat_ALP1.C02 ttggaaaagatgctttagattaatctttaatctttcatctaatgatattgtaaaaatgtc

goat_GRS.A04 ttggaaaagatgctttagattaatcttaaatctttcatcttatgatattgtaaaaatgtc

++ intron 2 ++++++++++++++++++++++++++++++++++++++++++++++++

DRB*1_GR0150 ttttcctcaaatttca--tttttttattgaacctgttagctttagggattgtatcaggca

DRB*1_GR0701 ttttcctcaaatttca--tttttttattgaacctgttagctttagggattgtatcaggca

DRB*1_GR0721 ttttcctcaaatttca--tttttttattgaacctgttagctttagggattgtatcaggca

DRB*1_GR0732 ttttcctcaaatttca--tttttttattgaacctgttagctttagggattgtatcaggca

DRB*1_VD0030 ttttcctcaaatttca--tttttttattgaacctgttagctttagggattgtatcaggca

DRB*1_VS0112 ttttcctcaaatttca--tttttttattgaacctgttagctttagggattgtatcaggca

DRB*1_VS0139 ttttcctcaaatttca--tttttttattgaacctgttagctttagggattgtatcaggca

DRB*2_GR0023 ttttcctcagatttcatttttttttattgaacccattagctttagggattgtatcaggca

DRB*2_GR0034 ttttcctcagatttcatttttttttattgaacccattagctttagggattgtatcaggca

DRB*2_GR0065 ttttcctcagatttcatttttttttattgaacccattagctttagggattgtatcaggca

DRB*2_GR0140 ttttcctcagatttcatttttttttattgaacccattagctttagggattgtatcaggca

DRB*2_GR0201 ttttcctcagatttcatttttttttattgaacccattagctttagggattgtatcaggca

DRB*2_GR0310 ttttcctcagatttcatttttttttattgaacccattagctttagggattgtatcaggca

DRB*2_GR0616 ttttcctcagatttcatttttttttattgaacccattagctttagggattgtatcaggca

goat_VBN4 ttttcctcagatttcatttttttttattgaacccattagctttagggattgtatcaggca

goat_ALP1.F01 ttttcctcagatttaa--tttttttattgaacccattagctttagggattgtatcaggca

goat_ALP1.E02 ttttcctcagatttaa--tttttttattgaacccattagctttagggattgtatcaggca

goat_ALP1.C02 ttttcctcagatttca--tttttttattgaacctgttagctttagggattgtatcaggca

goat_GRS.A04 ttttcctcagatttca--tttttttattgaacctgttagctttagggattgtatcaggca

++ intron 2 ++++++++++++++++++++++++++++++++++++++++++++++++

DRB*1_GR0150 ctaagtatacaaattaatgagtctcccatcctctcttgaactcaaatatgagcttaaaaa

DRB*1_GR0701 ctaagtatacaaattaatgagtctcccatcctctcttgaactcaaatatgagcttaaaaa

DRB*1_GR0721 ctaagtatacaaattaatgagtctcccatcctctcttgaactcaaatatgagcttaaaaa

DRB*1_GR0732 ctaagtatacaaattaatgagtctcccatcctctcttgaactcaaatatgagcttaaaaa

DRB*1_VD0030 ctaagtatacaaattaatgagtctcccatcctctcttgaactcaaatatgagcttaaaaa

DRB*1_VS0112 ctaagtatacaaattaatgagtctcccatcctctcttgaactcaaatatgagcttaaaaa

DRB*1_VS0139 ctaagtatacaaattaatgagtctcccatcctctcttgaactcaaatatgagcttaaaaa

DRB*2_GR0023 ctaagtatacatattaatgagtctcctatcctctcttgaactcaaatatgagcttaaaaa

DRB*2_GR0034 ctaagtatacatattaatgagtctcctatcctctcttgaactcaaatatgagcttaaaaa

DRB*2_GR0065 ctaagtatacatattaatgagtctcctatcctctcttgaactcaaatatgagcttaaaaa

DRB*2_GR0140 ctaagtatacatattaatgagtctcctatcctctcttgaactcaaatatgagcttaaaaa

DRB*2_GR0201 ctaagtatacatattaatgagtctcctatcctctcttgaactcaaatatgagcttaaaaa

DRB*2_GR0310 ctaagtatacatattaatgagtctcctatcctctcttgaactcaaatatgagcttaaaaa

DRB*2_GR0616 ctaagtatacatattaatgagtctcctatcctctcttgaactcaaatatgagcttaaaaa

goat_VBN4 ctaagtatacatattaatgagtctcctatcctctcttgaactcaaatatgagcttaaaaa

goat_ALP1.F01 ctaagcatacatattaatgagtctcccatcttctcttgaactcaaatatgagcttaaaaa

goat_ALP1.E02 ctaagcatacatattaatgagtctcccatcttctcttgaactcaaatatgagcttaaaaa

goat_ALP1.C02 ctaagcatacatattaatgagtctcccatcctctcttgaagtcaaatatgagcttaaaaa

goat_GRS.A04 ctaagcatacatattaatgagtctcccatcctctcttgaagtcaaatatgancttaaaaa

++ intron 2 ++++++++++++++++++++++++++++++++++++++++++++++++

DRB*1_GR0150 gaaataaaaatgtaaaataataatagtgccaatagtaaatatgcttgttttgcaaaggag

DRB*1_GR0701 gaaataaaaatgtaaaataataatagtgccaatagtaaatatgcttgttttgcaaaggag

DRB*1_GR0721 gaaataaaaatgtaaaataataatagtgccaatagtaaatatgcttgttttgcaaaggag

DRB*1_GR0732 gaaataaaaatgtaaaataataatagtgccaatagtaaatatgcttgttttgcaaaggag

DRB*1_VD0030 gaaataaaaatgtaaaataataatagtgccaatagtaaatatgcttgttttgcaaaggag

DRB*1_VS0112 gaaataaaaatgtaaaataataatagtgccaatagtaaatatgcttgttttgcaaaggag

DRB*1_VS0139 gaaataaaaatgtaaaataataatagtgccaatagtaaatatgcttgttttgcaaaggag

DRB*2_GR0023 gaaagaaaaatgtaaaataataatagttccaatagtaaatatgcttgttttacaaaggag

DRB*2_GR0034 gaaagaaaaatgtaaaataataatagttccaatagtaaatatgcttgttttacaaaggag

DRB*2_GR0065 gaaagaaaaatgtaaaataataatagttccaatagtaaatatgcttgttttacaaaggag

DRB*2_GR0140 gaaagaaaaatgtaaaataataatagttccaatagtaaatatgcttgttttacaaaggag

DRB*2_GR0201 gaaagaaaaatgtaaaataataatagttccaatagtaaatatgcttgttttacaaaggag

DRB*2_GR0310 gaaagaaaaatgtaaaataataatagttccaatagtaaatatgcttgttttacaaaggag

DRB*2_GR0616 gaaagaaaaatgtaaaataataatagttccaatagtaaatatgcttgttttacaaaggag

goat_VBN4 gaaagaaaaatgtaaaataataatagttccaatagtaaatatgcttgttttacaaaggag

goat_ALP1.F01 gaaagaaaaatgtaaaataataatagttccaatagtatatatgcttgttttacaaaggag

goat_ALP1.E02 gaaagaaaaatgtaaaataataatagttccaatagtaaatatgcttgttttacaaaggag

goat_ALP1.C02 gaaagaaaaatataaaataataatagttccaatagtgaatatgcttgttttgcaaaggaa

goat_GRS.A04 gaaagaaaaatgtaaaataataatagttccaatagtgaatatgcttgttttgcaaaggaa

++ intron 2 +++++++++++++++++++++++++++++++++

DRB*1_GR0150 atgggttgatcagtggtatgtaagaatttacatacattaaaaaaa

DRB*1_GR0701 atgggttgatcagtggtatgtaagaatttacatacattaaaaaaa

DRB*1_GR0721 atgggttgatcagtggtatgtaagaatttacatacattaaaaaaa

DRB*1_GR0732 atgggttgatcagtggtatgtaagaatttacatacattaaaaaaa

DRB*1_VD0030 atgggttgatcagtggtatgtaagaatttacatacattaaaaaaa

DRB*1_VS0112 atgggttgatcagtggtatgtaagaatttacatacattaaaaaaa

DRB*1_VS0139 atgggttgatcagtggtatgtaagaatttacatacattaaaaaaa

DRB*2_GR0023 atgggttgatcagtggtatgtaagaatttatatacacttaaaaaa

DRB*2_GR0034 atgggttgatcagtggtatgtaagaatttatatacacttaaaaaa

DRB*2_GR0065 atgggttgatcagtggtatgtaagaatttatatacacttaaaaaa

DRB*2_GR0140 atgggttgatcagtggtatgtaagaatttatatacacttaaaaaa

DRB*2_GR0201 atgggttgatcagtggtatgtaagaatttatatacacttaaaaaa

DRB*2_GR0310 atgggttgatcagtggtatgtaagaatttatatacacttaaaaaa

DRB*2_GR0616 atgggttgatcagtggtatgtaagaatttatatacacttaaaaaa

goat_VBN4 atgggttgatcagtggtatgtaagaatttatatacacttaaaaaa

goat_ALP1.F01 atgggttgttcagtggtatgtaagaatttgtatacactttgaaaa

goat_ALP1.E02 atgggttgttcagtggtatgtaagaatttgtatacactttgaaaa

goat_ALP1.C02 atgggttgatcagtggtatgtaagaatttatatacatttaaaaaa

goat_GRS.A04 atgggttgatcagtggtatgtaagaatttatatacattaaaaaaa
