# Supplementary material for: Introgression from Domestic Goat Generated Variation at the Major Histocompatibility Complex of Alpine Ibex
Source: PLoS Genet. 2014 Jun 19;10(6):e1004438. doi: 10.1371/journal.pgen.1004438 (PMC4063738; doi:10.1371/journal.pgen.1004438)
Supplement: Text S3 — MHC DRB sequence alignment. Partial intron 2, exon 3, partial intron 3. (DOC) [file pgen.1004438.s017.doc]

**MHC DRB sequence alignment**

partial intron 2, exon 3, partial intron 3

++ intron 2 ++++++++++++++++++++++++++++++++++++++++++++++++

DRB*1_GR0150 tcaatatctttcaaactaatggtccagtgtgtaaatgttaaagacaaagcccatggcact

DRB*1_GR0701 tcaatatctttcaaactaatggtccagtgtgtaaatgttaaagacaaagcccatggcact

DRB*1_GR0721 tcaatatctttcaaactaatggtccagtgtgtaaatgttaaagacaaagcccatggcact

DRB*1_GR0732 tcaatatctttcaaactaatggtccagtgtgtaaatgttaaagacaaagcccatggcact

DRB*1_VD0030 tcaatatctttcaaactaatggtccagtgtgtaaatgttaaagacaaagcccatggcact

DRB*1_VS0112 tcaatatctttcaaactaatggtccagtgtgtaaatgttaaagacaaagcccatggcact

DRB*1_VS0139 tcaatatctttcaaactaatggtccagtgtgtaaatgttaaagacaaagcccatggcact

DRB*2_GR0023 tcaacatctttcaaactaatggtccggtgtgtaaatgttaaacataaagctcatggcatt

DRB*2_GR0034 tcaacatctttcaaactaatggtccggtgtgtaaatgttaaacataaagctcatggcatt

DRB*2_GR0065 tcaacatctttcaaactaatggtccggtgtgtaaatgttaaacataaagctcatggcatt

DRB*2_GR0140 tcaacatctttcaaactaatggtccggtgtgtaaatgttaaacataaagctcatggcatt

DRB*2_GR0201 tcaacatctttcaaactaatggtccggtgtgtaaatgttaaacataaagctcatggcatt

DRB*2_GR0310 tcaacatctttcaaactaatggtccggtgtgtaaatgttaaacataaagctcatggcatt

DRB*2_GR0616 tcaacatctttcaaactaatggtccggtgtgtaaatgttaaacataaagctcatggcatt

goat_VBN4 tcaacatctttcaaactaatggtccggtgtgtaaatgttaaacataaagctcatggcatt

goat_ALP1.F01 tcaacatctttcaaactaat-gtccaatgtgtaaatgttaaagataaagctcatggcact

goat_ALP1.E02 tcaacatctttcaaactaat-gtccaatgtgtaaatgttaaagataaagctcatggcact

goat_ALP1.C02 tcaacatctttcaaantaatggtccagtgtgtaaatnttaaanataangctcatggcact

goat_GRS.A04 tcaacatctttcaaactaatggtccagtgtgtaaatgttaaagataaagctcatggcact

++ intron 2 ++++++++++++++++++++++++++++++++++++++++++++++++

DRB*1_GR0150 aattcctcttgtgccactgggccagctatgagagaggcctacccacggctgcggtctgga

DRB*1_GR0701 aattcctcttgtgccactgggccagctatgagagaggcctacccacggctgcggtctgga

DRB*1_GR0721 aattcctcttgtgccactgggccagctatgagagaggcctacccacggctgcggtctgga

DRB*1_GR0732 aattcctcttgtgccactgggccagctatgagagaggcctacccacggctgcggtctgga

DRB*1_VD0030 aattcctcttgtgccactgggccagctatgagagaggcctacccacggctgcggtctgga

DRB*1_VS0112 aattcctcttgtgccactgggccagctatgagagaggcctacccacggctgcggtctgga

DRB*1_VS0139 aattcctcttgtgccactgggccagctatgagagaggcctacccacggctgcggtctgga

DRB*2_GR0023 aattcctcttgtgccactg----------------ggcttacccaaggctacagtctgga

DRB*2_GR0034 aattcctcttgtgccactg----------------ggcttacccaaggctacagtctgga

DRB*2_GR0065 aattcctcttgtgccactg----------------ggcttacccaaggctacagtctgga

DRB*2_GR0140 aattcctcttgtgccactg----------------ggcttacccaaggctacagtctgga

DRB*2_GR0201 aattcctcttgtgccactg----------------ggcttacccaaggctacagtctgga

DRB*2_GR0310 aattcctcttgtgccactg----------------ggcttacccaaggctacagtctgga

DRB*2_GR0616 aattcctcttgtgccactg----------------ggcttacccaaggctacagtctgga

goat_VBN4 aattcctcttgtgccactg----------------ggcttacccaaggctacagtctgga

goat_ALP1.F01 aattcctcttgtgccactgggccagctgtaagggaggcctacccaaggctgcagtct-ga

goat_ALP1.E02 aattcctcttgtgccactgggccagctgtgagggaggcctacccaaggctgcagtct-ga

goat_ALP1.C02 aattcctcttgtgccactgggccagntgtgagggaggcctacnnnaggctgcagtctgga

goat_GRS.A04 aattcctcttgttccactgngccagctgtgagggaggcctacccaaggctgcagtctgga

++ intron 2 ++++++++++++++++++++++++++++++++++++++++++++++++

DRB*1_GR0150 actttagagtgggggctgaaaggcagctaaccaaggagacttactctgttgtccgcacta

DRB*1_GR0701 actttagagtgggggctgaaaggcagctaaccaaggagacttactctgttgtccgcacta

DRB*1_GR0721 actttagagtgggggctgaaaggcagctaaccaaggagacttactctgttgtccgcacta

DRB*1_GR0732 actttagagtgggggctgaaaggcagctaaccaaggagacttactctgttgtccgcacta

DRB*1_VD0030 actttagagtgggggctgaaaggcagctaaccaaggagacttactctgttgtccgcacta

DRB*1_VS0112 actttagagtgggggctgaaaggcagctaaccaaggagacttactctgttgtccgcacta

DRB*1_VS0139 actttagagtgggggctgaaaggcagctaaccaaggagacttactctgttgtccgcacta

DRB*2_GR0023 acttgagagtaggggctgaaaggcagctaaccaaggagacttactctgttgtcctcacta

DRB*2_GR0034 acttgagagtaggggctgaaaggcagctaaccaaggagacttactctgttgtcctcacta

DRB*2_GR0065 acttgagagtaggggctgaaaggcagctaaccaaggagacttactctgttgtcctcacta

DRB*2_GR0140 acttgagagtaggggctgaaaggcagctaaccaaggagacttactctgttgtcctcacta

DRB*2_GR0201 acttgagagtaggggctgaaaggcagctaaccaaggagacttactctgttgtcctcacta

DRB*2_GR0310 acttgagagtaggggctgaaaggcagctaaccaaggagacttactctgttgtcctcacta

DRB*2_GR0616 acttgagagtaggggctgaaaggcagctaaccaaggagacttactctgttgtcctcacta

goat_VBN4 acttgagagtaggggctgaaaggcagctaaccaaggaaacttactctgttgtcctcacta

goat_ALP1.F01 acttgagagtgggggctgaaaggcagctaaccaaggagacttactctgttgtcctcactg

goat_ALP1.E02 acttgagagtgggggctgaaaggcagctaaccaaggagacttactctgttgtcctcactg

goat_ALP1.C02 acttgagagtgrgggctgaaaggcagctaaccaaggaaacttactctgttgtcttcactg

goat_GRS.A04 acttgagantgagggctgaaaggcagctaaccaaggaaacttactntgttgtcttcactg

++ intron 2 +++++++++++++** exon 3 *************************

DRB*1_GR0150 attgcctccaccttttctctcccagtggagcctatagtgactgtgtatcctgcgaagacc

DRB*1_GR0701 attgcctccaccttttctctcccagtggagcctatagtgactgtgtatcctgcgaagacc

DRB*1_GR0721 attgcctccaccttttctctcccagtggagcctatagtgactgtgtatcctgcgaagacc

DRB*1_GR0732 attgcctccaccttttctctcccagtggagcctatagtgactgtgtatcctgcgaagacc

DRB*1_VD0030 attgcctccaccttttctctcccagtggagcctatagtgactgtgtatcctgcgaagacc

DRB*1_VS0112 attgcctccaccttttctctcccagtggagcctatagtgactgtgtatcctgcgaagacc

DRB*1_VS0139 attgcctccaccttttctctcccagtggagcctatagtgactgtgtatcctgcgaagacc

DRB*2_GR0023 actgcctccaccttttctctcccagtggagcctgcagtgactgtgtatcctacaaagacc

DRB*2_GR0034 actgcctccaccttttctctcccagtggagcctgcagtgactgtgtatcctacaaagacc

DRB*2_GR0065 actgcctccaccttttctctcccagtggagcctgcagtgactgtgtatcctacaaagacc

DRB*2_GR0140 actgcctccaccttttctctcccagtggagcctgcagtgactgtgtatcctacaaagacc

DRB*2_GR0201 actgcctccaccttttctctcccagtggagcctgcagtgactgtgtatcctacaaagacc

DRB*2_GR0310 actgcctccaccttttctctcccagtggagcctgcagtgactgtgtatcctacaaagacc

DRB*2_GR0616 actgcctccaccttttctctcccagtggagcctgcagtgactgtgtatcctacaaagacc

goat_VBN4 actgcctccaccttttctctcccagtggagcctgcagtgactgtgtatcctgcaaagacc

goat_ALP1.F01 atttcctccaccttttccctcctagtggagcctacagtgactgtgtatcctgcaaagacc

goat_ALP1.E02 atttcctccaccttttccctcctagtggagcctacagtgactgtgtatcctgcaaagacc

goat_ALP1.C02 atytcctccaccttttccctcctagtggagcctatagtgactgtgtatcctgcaaagacc

goat_GRS.A04 atctcctccaccttttccctcctagtggagcctacagtgactgtgtatcctgcaaagacc

** exon 3 **************************************************

DRB*1_GR0150 cagcccctgcagcaccacaacctcctggtctgctctgtgaacggattctacccaggccac

DRB*1_GR0701 cagcccctgcagcaccacaacctcctggtctgctctgtgaacggattctacccaggccac

DRB*1_GR0721 cagcccctgcagcaccacaacctcctggtctgctctgtgaacggattctacccaggccac

DRB*1_GR0732 cagcccctgcagcaccacaacctcctggtctgctctgtgaacggattctacccaggccac

DRB*1_VD0030 cagcccctgcagcaccacaacctcctggtctgctctgtgaacggattctacccaggccac

DRB*1_VS0112 cagcccctgcagcaccacaacctcctggtctgctctgtgaacggattctacccaggccac

DRB*1_VS0139 cagcccctgcagcaccacaacctcctggtctgctctgtgaacggattctacccaggccac

DRB*2_GR0023 cagcccctgcagcaccacaacctcctggtctgctctgtgaatggattctacccaggccac

DRB*2_GR0034 cagcccctgcagcaccacaacctcctggtctgctctgtgaatggattctacccaggccac

DRB*2_GR0065 cagcccctgcagcaccacaacctcctggtctgctctgtgaatggattctacccaggccac

DRB*2_GR0140 cagcccctgcagcaccacaacctcctggtctgctctgtgaatggattctacccaggccac

DRB*2_GR0201 cagcccctgcagcaccacaacctcctggtctgctctgtgaatggattctacccaggccac

DRB*2_GR0310 cagcccctgcagcaccacaacctcctggtctgctctgtgaatggattctacccaggccac

DRB*2_GR0616 cagcccctgcagcaccacaacctcctggtctgctctgtgaatggattctacccaggccac

goat_VBN4 cagcccctgcagcaccacaacctcctggtctgctctgtgaatggattctacccaggccac

goat_ALP1.F01 cagcccctgcagcaccacaacctcctggtctgctctgtgaatggattctacccaggccac

goat_ALP1.E02 cagcccctgcagcaccacaacctcctggtctgctctgtgaatggattctacccaggccac

goat_ALP1.C02 cagcccctgcagcaccacaacctcctggtctgctctgtgaatggattctacccaggccac

goat_GRS.A04 cagcccctgcagcaccacaacctcctggtctgctctgtgaatggattctacccaggccac

** exon 3 **************************************************

DRB*1_GR0150 attgaagtcaggtggttccgaaatggccatgaagaggaggctggggtgatctccacaggc

DRB*1_GR0701 attgaagtcaggtggttccgaaatggccatgaagaggaggctggggtgatctccacaggc

DRB*1_GR0721 attgaagtcaggtggttccgaaatggccatgaagaggaggctggggtgatctccacaggc

DRB*1_GR0732 attgaagtcaggtggttccgaaatggccatgaagaggaggctggggtgatctccacaggc

DRB*1_VD0030 attgaagtcaggtggttccgaaatggccatgaagaggaggctggggtgatctccacaggc

DRB*1_VS0112 attgaagtcaggtggttccgaaatggccatgaagaggaggctggggtgatctccacaggc

DRB*1_VS0139 attgaagtcaggtggttccgaaatggccatgaagaggaggctggggtgatctccacaggc

DRB*2_GR0023 attgaagtcaggtggttccggaacggccacgaagaggaggctggggtgatctccacaggc

DRB*2_GR0034 attgaagtcaggtggttccggaacggccacgaagaggaggctggggtgatctccacaggc

DRB*2_GR0065 attgaagtcaggtggttccggaacggccacgaagaggaggctggggtgatctccacaggc

DRB*2_GR0140 attgaagtcaggtggttccggaacggccacgaagaggaggctggggtgatctccacaggc

DRB*2_GR0201 attgaagtcaggtggttccggaacggccacgaagaggaggctggggtgatctccacaggc

DRB*2_GR0310 attgaagtcaggtggttccggaacggccacgaagaggaggctggggtgatctccacaggc

DRB*2_GR0616 attgaagtcaggtggttccggaacggccacgaagaggaggctggggtgatctccacaggc

goat_VBN4 attgaagtcaggtggttccggaacggccacgaagaggaggctggggtgatctccacaggc

goat_ALP1.F01 attgaagtcaggtggttccggaacggccacgaagaggaggctggggtgatctccacaggc

goat_ALP1.E02 attgaagtcaggtggttccggaacggccacgaagaggaggctggggtgatctccacaggc

goat_ALP1.C02 attgaagtcaggtggttccggaacrgccaygaagaggaggctggggtgatctccacaggc

goat_GRS.A04 attgaagtcaggtggttccggaacagccatgaagaggaggctggagtgatctccacaggc

** exon 3 **************************************************

DRB*1_GR0150 ctgatccagaatggagactggaccttccagaccatggtgatgcttgaaacagttcctcag

DRB*1_GR0701 ctgatccagaatggagactggaccttccagaccatggtgatgcttgaaacagttcctcag

DRB*1_GR0721 ctgatccagaatggagactggaccttccagaccatggtgatgcttgaaacagttcctcag

DRB*1_GR0732 ctgatccagaatggagactggaccttccagaccatggtgatgcttgaaacagttcctcag

DRB*1_VD0030 ctgatccagaatggagactggaccttccagaccatggtgatgcttgaaacagttcctcag

DRB*1_VS0112 ctgatccagaatggagactggaccttccagaccatggtgatgcttgaaacagttcctcag

DRB*1_VS0139 ctgatccagaatggagactggaccttccagaccatggtgatgcttgaaacagttcctcag

DRB*2_GR0023 ctgatccagaatggagactggaccttccagaccatggtgatgcttgaaacagttcctcag

DRB*2_GR0034 ctgatccagaatggagactggaccttccagaccatggtgatgcttgaaacagttcctcag

DRB*2_GR0065 ctgatccagaatggagactggaccttccagaccatggtgatgcttgaaacagttcctcag

DRB*2_GR0140 ctgatccagaatggagactggaccttccagaccatggtgatgcttgaaacagttcctcag

DRB*2_GR0201 ctgatccagaatggagactggaccttccagaccatggtgatgcttgaaacagttcctcag

DRB*2_GR0310 ctgatccagaatggagactggaccttccagaccatggtgatgcttgaaacagttcctcag

DRB*2_GR0616 ctgatccagaatggagactggaccttccagaccatggtgatgcttgaaacagttcctcag

goat_VBN4 ctgatccagaatggagactggaccttccagaccatggtgatgcttgaaacagttcctcag

goat_ALP1.F01 ctgatccagaatggagactggaccttccagaccatggtgatgcttgaaacagttcctcag

goat_ALP1.E02 ctgatccagaatggagactggaccttccagaccatggtgatgcttgaaacagttcctcag

goat_ALP1.C02 ctgatccagaatggagactggaccttccagaccatggtgatgcttgaaacagttcctcag

goat_GRS.A04 ctgatccagaatggagactggaccttccagaccatggtgatgcttgaaacagttcctcan

** exon 3 **************************************************

DRB*1_GR0150 agtggagaggtctatacctgccaagtggagcaccccagccggacgagccctatcacagta

DRB*1_GR0701 agtggagaggtctatacctgccaagtggagcaccccagccggacgagccctatcacagta

DRB*1_GR0721 agtggagaggtctatacctgccaagtggagcaccccagccggacgagccctatcacagta

DRB*1_GR0732 agtggagaggtctatacctgccaagtggagcaccccagccggacgagccctatcacagta

DRB*1_VD0030 agtggagaggtctatacctgccaagtggagcaccccagccggacgagccctatcacagta

DRB*1_VS0112 agtggagaggtctatacctgccaagtggagcaccccagccggacgagccctatcacagta

DRB*1_VS0139 agtggagaggtctatacctgccaagtggagcaccccagccggacgagccctatcacagta

DRB*2_GR0023 ggtggagaggtctacacctgccaagtggagcaccccagccggacgagccctatcacagta

DRB*2_GR0034 ggtggagaggtctacacctgccaagtggagcaccccagccggacgagccctatcacagta

DRB*2_GR0065 ggtggagaggtctacacctgccaagtggagcaccccagccggacgagccctatcacagta

DRB*2_GR0140 ggtggagaggtctacacctgccaagtggagcaccccagccggacgagccctatcacagta

DRB*2_GR0201 ggtggagaggtctacacctgccaagtggagcaccccagccggacgagccctatcacagta

DRB*2_GR0310 ggtggagaggtctacacctgccaagtggagcaccccagccggacgagccctatcacagta

DRB*2_GR0616 ggtggagaggtctacacctgccaagtggagcaccccagccggacgagccctatcacagta

goat_VBN4 ggtggagaggtctacacctgccaagtggagcaccccagccggacgagccctatcacagta

goat_ALP1.F01 agtggagaggtctacacctgccaagtggagcaccccagccggacgagccctatcacagta

goat_ALP1.E02 agtggagaggtctacacctgccaagtggancaccccagccggacgagccctatcacagta

goat_ALP1.C02 agtggagaggtctacacctgccaagtggagcaccccagccggacgagccctatcacagtn

goat_GRS.A04 agtggagaggtctacacctgccaagtggagcaccccagccggacgagccctatcacagta

*******++ intron 3 +++++++++++++++++++++++++++++++++++++++++

DRB*1_GR0150 gaatggagtgagctttctgatctcataaatccctcacccaccgtggagggggcttgcttt

DRB*1_GR0701 gaatggagtgagctttctgatctcataaatccctcacccaccgtggagggggcttgcttt

DRB*1_GR0721 gaatggagtgagctttctgatctcataaatccctcacccaccgtggagggggcttgcttt

DRB*1_GR0732 gaatggagtgagctttctgatctcataaatccctcacccaccgtggagggggcttgcttt

DRB*1_VD0030 gaatggagtgagctttctgatctcataaatccctcacccaccgtggagggggcttgcttt

DRB*1_VS0112 gaatggagtgagctttctgatctcataaatccctcacccaccgtggagggggcttgcttt

DRB*1_VS0139 gaatggagtgagctttctgatctcataaatccctcacccaccgtggagggggcttgcttt

DRB*2_GR0023 gaatggagtgagctttctgatctcataaatccctcacccaccgtggagggggcttgcttt

DRB*2_GR0034 gaatggagtgagctttctgatctcataaatccctcacccaccgtggagggggcttgcttt

DRB*2_GR0065 gaatggagtgagctttctgatctcataaatccctcacccaccgtggagggggcttgcttt

DRB*2_GR0140 gaatggagtgagctttctgatctcataaatccctcacccaccgtggagggggcttgcttt

DRB*2_GR0201 gaatggagtgagctttctgatctcataaatccctcacccaccgtggagggggcttgcttt

DRB*2_GR0310 gaatggagtgagctttctgatctcataaatccctcacccaccgtggagggggcttgcttt

DRB*2_GR0616 gaatggagtgagctttctgatctcataaatccctcacccaccgtggagggggcttgcttt

goat_VBN4 gaatggagtgagctttctgatctcataaatccctcacccaccgtggagggggcttgcttt

goat_ALP1.F01 gaatggagtgagctttctgatctcataaatccctcacccaccgtggagggggcttgcttt

goat_ALP1.E02 gaatggagtgagctttctgatctcataaatccctcacccaccgtggagggggcttgcttt

goat_ALP1.C02 gaatggagtgagctttctgatctcacaaatccctcacccaccgtggagggggcttgcttt

goat_GRS.A04 gaatggagtgagctttctgatctcacaaatccctcacccaccgtggagggggcttgcttt

++ intron 3 ++++++++++++++++++++++++++++++++++++++++++++++++

DRB*1_GR0150 cccctgagtgtcccctgagtgtcttctctccctacaccatgtgttcatttgctttttgtt

DRB*1_GR0701 cccctgagtgtcccctgagtgtcttctctccctacaccatgtgttcatttgctttttgtt

DRB*1_GR0721 cccctgagtgtcccctgagtgtcttctctccctacaccatgtgttcatttgctttttgtt

DRB*1_GR0732 cccctgagtgtcccctgagtgtcttctctccctacaccatgtgttcatttgctttttgtt

DRB*1_VD0030 cccctgagtgtcccctgagtgtcttctctccctacaccatgtgttcatttgctttttgtt

DRB*1_VS0112 cccctgagtgtcccctgagtgtcttctctccctacaccatgtgttcatttgctttttgtt

DRB*1_VS0139 cccctgagtgtcccctgagtgtcttctctccctacaccatgtgttcatttgctttttgtt

DRB*2_GR0023 cccctgagcgtcccctgagtgtctcctctccctacaccatgtgttcatttgctccttgtt

DRB*2_GR0034 cccctgagcgtcccctgagtgtctcctctccctacaccatgtgttcatttgctccttgtt

DRB*2_GR0065 cccctgagcgtcccctgagtgtctcctctccctacaccatgtgttcatttgctccttgtt

DRB*2_GR0140 cccctgagcgtcccctgagtgtctcctctccctacaccatgtgttcatttgctccttgtt

DRB*2_GR0201 cccctgagcgtcccctgagtgtctcctctccctacaccatgtgttcatttgctccttgtt

DRB*2_GR0310 cccctgagcgtcccctgagtgtctcctctccctacaccatgtgttcatttgctccttgtt

DRB*2_GR0616 cccctgagcgtcccctgagtgtctcctctccctacaccatgtgttcatttgctccttgtt

goat_VBN4 cccctgagcgtcccctgagtgtctcctctccctacaccatgtgttcatttgctccttgtt

goat_ALP1.F01 cccctgagcgtcccctgagtgtctcctctccctacaccatgtgttcatttgctccttgtt

goat_ALP1.E02 cccctgaNNNNNNNNNNNNNNNNNNNNNNNNNNNNNNNNNNNNNNNNNNNNNNNNNNNNN

goat_ALP1.C02 cccctgagtgtcccctgantgtntcctctccntacaccatgtgttcatttgctccttgtt

goat_GRS.A04 cccctgagtgtcccctgagtgtctcctctccctacaccatgtgttcatttgctccttgtt
