# Supplementary material for: Introgression from Domestic Goat Generated Variation at the Major Histocompatibility Complex of Alpine Ibex
Source: PLoS Genet. 2014 Jun 19;10(6):e1004438. doi: 10.1371/journal.pgen.1004438 (PMC4063738; doi:10.1371/journal.pgen.1004438)
Supplement: Text S4 — MHC DRB sequence alignment. Partial intron 4, exon 5, intron 5, exon 6, 3′ UTR. (DOC) [file pgen.1004438.s018.doc]

**MHC DRB sequence alignment**

partial intron 4, exon 5, intron 5, exon 6, 3' UTR

++ intron 4 ++++++++++++++++++++++++++++++++++++++++++++++++

DRB*1_GR0150 tgaaagaaacttcatgggtcacatgttctcagtgtttgccttcgtataaaccctggcatc

DRB*1_GR0701 tgaaagaaacttcatgggtcacatgttctcagtgtttgccttcgtataaaccctggcatc

DRB*1_GR0721 tgaaagaaacttcatgggtcacatgttctcagtgtttgccttcgtataaaccctggcatc

DRB*1_GR0732 tgaaagaaacttcatgggtcacatgttctcagtgtttgccttcgtataaaccctggcatc

DRB*1_VD0030 tgaaagaaacttcatgggtcacatgttctcagtgtttgccttcgtataaaccctggcatc

DRB*1_VS0112 tgaaagaaacttcatgggtcacatgttctcagtgtttgccttcgtataaaccctggcatc

DRB*1_VS0139 tgaaagaaacttcatgggtcacatgttctcagtgtttgccttcgtataaaccctggcatc

DRB*2_GR0023 tgagagaaacttcattggtcacatgctgtcagtgtttgccttcatataaaccctgaaatc

DRB*2_GR0034 tgagagaaacttcattggtcacatgctgtcagtgtttgccttcatataaaccctgaaatc

DRB*2_GR0065 tgagagaaacttcattggtcacatgctgtcagtgtttgccttcatataaaccctgaaatc

DRB*2_GR0140 tgagagaaacttcattggtcacatgctgtcagtgtttgccttcatataaaccctgaaatc

DRB*2_GR0201 tgagagaaacttcattggtcacatgctgtcagtgtttgccttcatataaaccctgaaatc

DRB*2_GR0310 tgagagaaacttcattggtcacatgctgtcagtgtttgccttcatataaaccctgaaatc

DRB*2_GR0616 tgagagaaacttcattggtcacatgctgtcagtgtttgccttcatataaaccctgaaatc

goat_VBN4 tgagagaaacttcattggtcacatgctgtcagtgtttgccttcatataaaccctgaaatc

goat_ALP1.F01 tgagagaaacttcattggtcacatgctgtcagtgtttgccttcatataaaccctgaaatc

goat_ALP1.C02 tgagagaaacttcattggtcacatgctgtcagtgtttgccctcatataaaccctgaaatc

goat_GRS.A04 tgagagaaacttcatkggtcacatgctgtcagtgtttgccytcatataaaccctgaaatc

++ intron 4 ++++++++++++++++++++++++++++++++++++++++++++++++

DRB*1_GR0150 agatgatgtg----------ttaggaggatctctggtggggccctggggcttggggcatt

DRB*1_GR0701 agatgatgtg----------ttaggaggatctctggtggggccctggggcttggggcatt

DRB*1_GR0721 agatgatgtg----------ttaggaggatctctggtggggccctggggcttggggcatt

DRB*1_GR0732 agatgatgtg----------ttaggaggatctctggtggggccctggggcttggggcatt

DRB*1_VD0030 agatgatgtg----------ttaggaggatctctggtggggccctggggcttggggcatt

DRB*1_VS0112 agatgatgtg----------ttaggaggatctctggtggggccctggggcttggggcatt

DRB*1_VS0139 agatgatgtg----------ttaggaggatctctggtggggccctggggcttggggcatt

DRB*2_GR0023 agatgatatgggttatggtattaggaggatctctggtggggccctggggcttaggacatt

DRB*2_GR0034 agatgatatgggttatggtattaggaggatctctggtggggccctggggcttaggacatt

DRB*2_GR0065 agatgatatgggttatggtattaggaggatctctggtggggccctggggcttaggacatt

DRB*2_GR0140 agatgatatgggttatggtattaggaggatctctggtggggccctggggcttaggacatt

DRB*2_GR0201 agatgatatgggttatggtattaggaggatctctggtggggccctggggcttaggacatt

DRB*2_GR0310 agatgatatgggttatggtattaggaggatctctggtggggccctggggcttaggacatt

DRB*2_GR0616 agatgatatgggttatggtattaggaggatctctggtggggccctggggcttaggacatt

goat_VBN4 agatgatatgggttatggtattaggaggatctctggtggggccctggggcttaggacatt

goat_ALP1.F01 agatgatatgggttatggtattaggaggatctctggtggggccctggggcttaggacatt

goat_ALP1.C02 agatgatatgggttatggtattaggaagatctcgggtggggccccggggcttgggacatt

goat_GRS.A04 agatgatatgggttatggtattaggaagatctcgggtggggccccggggcttgggacatt

++ intron 4 ++++++++++++++++++++++++++++++++++++++++++++++++

DRB*1_GR0150 gtgttttgggggtggatcaacctctatatcttttaagtatatatcttcttttgtctctcc

DRB*1_GR0701 gtgttttgggggtggatcaacctctatatcttttaagtatatatcttcttttgtctctcc

DRB*1_GR0721 gtgttttgggggtggatcaacctctatatcttttaagtatatatcttcttttgtctctcc

DRB*1_GR0732 gtgttttgggggtggatcaacctctatatcttttaagtatatatcttcttttgtctctcc

DRB*1_VD0030 gtgttttgggggtggatcaacctctatatcttttaagtatatatcttcttttgtctctcc

DRB*1_VS0112 gtgttttgggggtggatcaacctctatatcttttaagtatatatcttcttttgtctctcc

DRB*1_VS0139 gtgttttgggggtggatcaacctctatatcttttaagtatatatcttcttttgtctctcc

DRB*2_GR0023 gtgttttggggatggatcaacctctatatcttttaagtatatatcttcttttgtctctcc

DRB*2_GR0034 gtgttttggggatggatcaacctctatatcttttaagtatatatcttcttttgtctctcc

DRB*2_GR0065 gtgttttggggatggatcaacctctatatcttttaagtatatatcttcttttgtctctcc

DRB*2_GR0140 gtgttttggggatggatcaacctctatatcttttaagtatatatcttcttttgtctctcc

DRB*2_GR0201 gtgttttggggatggatcaacctctatatcttttaagtatatatcttcttttgtctctcc

DRB*2_GR0310 gtgttttggggatggatcaacctctatatcttttaagtatatatcttcttttgtctctcc

DRB*2_GR0616 gtgttttggggatggatcaacctctatatcttttaagtatatatcttcttttgtctctcc

goat_VBN4 gtgttttggggatggatcaacctctatatcttttaagtatatatcttcttttgtctctcc

goat_ALP1.F01 gtgttttggggatggatcaacctctatatcttttaagtatatatcttcttttgtctctcc

goat_ALP1.C02 gtgttttggggatggatcaacctctatatcttttaagtgtatatcttcttttgtctctcc

goat_GRS.A04 gtgttttggggatggatcaacctctatatcttttaagtgtatatcttcttttgtctctcc

+++** exon 5 **************++ intron 5 +++++++++++++++++++++

DRB*1_GR0150 taggacgccctacacttcagccaacaggtatgctctttatcttttagaatagtgtttagt

DRB*1_GR0701 taggacgccctacacttcagccaacaggtatgctctttatcttttagaatagtgtttagt

DRB*1_GR0721 taggacgccctacacttcagccaacaggtatgctctttatcttttagaatagtgtttagt

DRB*1_GR0732 taggacgccctacacttcagccaacaggtatgctctttatcttttagaatagtgtttagt

DRB*1_VD0030 taggacgccctacacttcagccaacaggtatgctctttatcttttagaatagtgtttagt

DRB*1_VS0112 taggacgccctacacttcagccaacaggtatgctctttatcttttagaatagtgtttagt

DRB*1_VS0139 taggacgccctacacttcagccaacaggtatgctctttatcttttagaatagtgtttagt

DRB*2_GR0023 taggacgccctacccttcagccaacaggtatgcactttatcttttagaatcgtgtttagt

DRB*2_GR0034 taggacgccctacccttcagccaacaggtatgcactttatcttttagaatcgtgtttagt

DRB*2_GR0065 taggacgccctacccttcagccaacaggtatgcactttatcttttagaatcgtgtttagt

DRB*2_GR0140 taggacgccctacccttcagccaacaggtatgcactttatcttttagaatcgtgtttagt

DRB*2_GR0201 taggacgccctacccttcagccaacaggtatgcactttatcttttagaatcgtgtttagt

DRB*2_GR0310 taggacgccctacccttcagccaacaggtatgcactttatcttttagaatcgtgtttagt

DRB*2_GR0616 taggacgccctacccttcagccaacaggtatgcactttatcttttagaatcgtgtttagt

goat_VBN4 taggacgccctacacttcagccaacaggtatgctctttatcttttagaatcgtgtttagt

goat_ALP1.F01 taggacgccctacacttcagccaacaggtatgctctttatcttttagaatcgtgtttagt

goat_ALP1.C02 taggacgccctacccttcagccaacaggtatgctctttatcttttagaatcatgtttagt

goat_GRS.A04 taggacgccctacmcttcagccaacaggtatgcwctttatcttttagaatcatgtttagt

++ intron 5 ++++++++++++++++++++++++++++++++++++++++++++++++

DRB*1_GR0150 ctccctggaacagatggtagaggtaacaagacagaggcagaaataatgaaagactttaga

DRB*1_GR0701 ctccctggaacagatggtagaggtaacaagacagaggcagaaataatgaaagactttaga

DRB*1_GR0721 ctccctggaacagatggtagaggtaacaagacagaggcagaaataatgaaagactttaga

DRB*1_GR0732 ctccctggaacagatggtagaggtaacaagacagaggcagaaataatgaaagactttaga

DRB*1_VD0030 ctccctggaacagatggtagaggtaacaagacagaggcagaaataatgaaagactttaga

DRB*1_VS0112 ctccctggaacagatggtagaggtaacaagacagaggcagaaataatgaaagactttaga

DRB*1_VS0139 ctccctggaacagatggtagaggtaacaagacagaggcagaaataatgaaagactttaga

DRB*2_GR0023 ctccctggaa--gatggtggaggaaacaagacagaggcagaaataatgaaagactttgga

DRB*2_GR0034 ctccctggaa--gatggtggaggaaacaagacagaggcagaaataatgaaagactttgga

DRB*2_GR0065 ctccctggaa--gatggtggaggaaacaagacagaggcagaaataatgaaagactttgga

DRB*2_GR0140 ctccctggaa--gatggtggaggaaacaagacagaggcagaaataatgaaagactttgga

DRB*2_GR0201 ctccctggaa--gatggtggaggaaacaagacagaggcagaaataatgaaagactttgga

DRB*2_GR0310 ctccctggaa--gatggtggaggaaacaagacagaggcagaaataatgaaagactttgga

DRB*2_GR0616 ctccctggaa--gatggtggaggaaacaagacagaggcagaaataatgaaagactttgga

goat_VBN4 ctccctggaa--gatggtggaggaaacaagacagaggca-------------actttgga

goat_ALP1.F01 ctccctggaa--gatggtggaggaaacaagacagaggcagaaataatgaaagactttgga

goat_ALP1.C02 ctccctggaacagatgatagagg--------------cagaaataatgaaagactttgga

goat_GRS.A04 ctccctggaacagatgatagagg--------------cagaaataatgaaagactttgga

++ intron 5 ++++++++++++++++++++++++++++++++++++++++++++++++

DRB*1_GR0150 cctgacttctcatcaggcagtttacactaaagcttcttcttgcaactaaataaaaggctt

DRB*1_GR0701 cctgacttctcatcaggcagtttacactaaagcttcttcttgcaactaaataaaaggctt

DRB*1_GR0721 cctgacttctcatcaggcagtttacactaaagcttcttcttgcaactaaataaaaggctt

DRB*1_GR0732 cctgacttctcatcaggcagtttacactaaagcttcttcttgcaactaaataaaaggctt

DRB*1_VD0030 cctgacttctcatcaggcagtttacactaaagcttcttcttgcaactaaataaaaggctt

DRB*1_VS0112 cctgacttctcatcaggcagtttacactaaagcttcttcttgcaactaaataaaaggctt

DRB*1_VS0139 cctgacttctcatcaggcagtttacactaaagcttcttcttgcaactaaataaaaggctt

DRB*2_GR0023 cctgacttctcatcaggcagtttacactaaagcttctt---tcaactaaataaaaggctt

DRB*2_GR0034 cctgacttctcatcaggcagtttacactaaagcttctt---tcaactaaataaaaggctt

DRB*2_GR0065 cctgacttctcatcaggcagtttacactaaagcttctt---tcaactaaataaaaggctt

DRB*2_GR0140 cctgacttctcatcaggcagtttacactaaagcttctt---tcaactaaataaaaggctt

DRB*2_GR0201 cctgacttctcatcaggcagtttacactaaagcttctt---tcaactaaataaaaggctt

DRB*2_GR0310 cctgacttctcatcaggcagtttacactaaagcttctt---tcaactaaataaaaggctt

DRB*2_GR0616 cctgacttctcatcaggcagtttacactaaagcttctt---tcaactaaataaaaggctt

goat_VBN4 cctgacttctcatcaggcagtttacactaaagcttctt---tcaactaaataaaaggctt

goat_ALP1.F01 cctgacttctcatcaggcagtttacactaaagcttctt---tcaactaaataaaaggctt

goat_ALP1.C02 cctgacttctcatcaggcagtttacactaaagcttcttctttcaactaaataaaaggctt

goat_GRS.A04 cctgacttctcatcaggcagtttacactaaagcntcttctttcaactaaataaaaggctt

++ intron 5 ++++++++++++++++++++++++++++++++++++++++++++++++

DRB*1_GR0150 atgctctaaagtagctttgcctcaggaaacttaagaatatttcccccttctaactgtaat

DRB*1_GR0701 atgctctaaagtagctttgcctcaggaaacttaagaatatttcccccttctaactgtaat

DRB*1_GR0721 atgctctaaagtagctttgcctcaggaaacttaagaatatttcccccttctaactgtaat

DRB*1_GR0732 atgctctaaagtagctttgcctcaggaaacttaagaatatttcccccttctaactgtaat

DRB*1_VD0030 atgctctaaagtagctttgcctcaggaaacttaagaatatttcccccttctaactgtaat

DRB*1_VS0112 atgctctaaagtagctttgcctcaggaaacttaagaatatttcccccttctaactgtaat

DRB*1_VS0139 atgctctaaagtagctttgcctcaggaaacttaagaatatttcccccttctaactgtaat

DRB*2_GR0023 gtgctctaaagtagctttgcctcaggaaacttaagaatattttccccttccaactgtaat

DRB*2_GR0034 gtgctctaaagtagctttgcctcaggaaacttaagaatattttccccttccaactgtaat

DRB*2_GR0065 gtgctctaaagtagctttgcctcaggaaacttaagaatattttccccttccaactgtaat

DRB*2_GR0140 gtgctctaaagtagctttgcctcaggaaacttaagaatattttccccttccaactgtaat

DRB*2_GR0201 gtgctctaaagtagctttgcctcaggaaacttaagaatattttccccttccaactgtaat

DRB*2_GR0310 gtgctctaaagtagctttgcctcaggaaacttaagaatattttccccttccaactgtaat

DRB*2_GR0616 gtgctctaaagtagctttgcctcaggaaacttaagaatattttccccttccaactgtaat

goat_VBN4 gtgctctaaagtagctttgcctcaggaaacttaagaatattttccccttccaactgtaat

goat_ALP1.F01 gtgctctaaagtagctttgcctcaggaaacttaagaatattttccccttccaactgtaat

goat_ALP1.C02 gtgctctaaagaagctttggctcaggaaactgaagaatattttccccttccaactgtaat

goat_GRS.A04 gtgcnctaaagtanctttggctcaggaaactgaagaatattttccccttccaactgtaat

++ intron 5 ++++++++++++++++++++++++++++++++++++++++++++++++

DRB*1_GR0150 gctttaatattaacattaacattcccctgtagagttatgtctggaaacaatcctctcctc

DRB*1_GR0701 gctttaatattaacattaacattcccctgtagagttatgtctggaaacaatcctctcctc

DRB*1_GR0721 gctttaatattaacattaacattcccctgtagagttatgtctggaaacaatcctctcctc

DRB*1_GR0732 gctttaatattaacattaacattcccctgtagagttatgtctggaaacaatcctctcctc

DRB*1_VD0030 gctttaatattaacattaacattcccctgtagagttatgtctggaaacaatcctctcctc

DRB*1_VS0112 gctttaatattaacattaacattcccctgtagagttatgtctggaaacaatcctctcctc

DRB*1_VS0139 gctttaatattaacattaacattcccctgtagagttatgtctggaaacaatcctctcctc

DRB*2_GR0023 gctttaatattaacattaacattcccctgtagagttatgtctggaaacaaccctctc--c

DRB*2_GR0034 gctttaatattaacattaacattcccctgtagagttatgtctggaaacaaccctctc--c

DRB*2_GR0065 gctttaatattaacattaacattcccctgtagagttatgtctggaaacaaccctctc--c

DRB*2_GR0140 gctttaatattaacattaacattcccctgtagagttatgtctggaaacaaccctctc--c

DRB*2_GR0201 gctttaatattaacattaacattcccctgtagagttatgtctggaaacaaccctctc--c

DRB*2_GR0310 gctttaatattaacattaacattcccctgtagagttatgtctggaaacaaccctctc--c

DRB*2_GR0616 gctttaatattaacattaacattcccctgtagagttatgtctggaaacaaccctctc--c

goat_VBN4 gctttaatattaacattaacattcccctgtagagttatgtctggaaacaaccctctc--c

goat_ALP1.F01 gctttaatattaacattaacattcccctgtagagttatgtctggaaacaaccctctc--c

goat_ALP1.C02 gctttaatattaacattaacattcccctgtagagttatgtctggaagcaaccctctcttc

goat_GRS.A04 gctttaatattaacattaacattcccctgtagagttatgtctggaagcaaccctctcytc

++++++++++++** exon 6 ****++ 3' UTR ++++++++++++++++++++++++

DRB*1_GR0150 tgtctcttgcagggctcctgagctgaagtgaagatggtcacactcaaggaagaaccttct

DRB*1_GR0701 tgtctcntgcagggctcntgagntgaagngaaganggtcacactcaaggaagaaccttct

DRB*1_GR0721 tgtctcttgcagggctcctgagctgaagtgaagatggtcacactcaaggaagaaccttct

DRB*1_GR0732 tgtctcttgcagggctcctgagctgaagtgaagatggtcacactcaaggaagaaccttct

DRB*1_VD0030 tgtctcntgcagggctcctgagctgaagtgaagatggtcacactcaaggaagaaccttct

DRB*1_VS0112 tgtctcttgcagggctcctgagctgaagtgaagatggtcacactcaaggaagaaccttct

DRB*1_VS0139 tgtctcntgcagggctcntgagntgaagtgaagatggtcacactcaaggaagaaccttct

DRB*2_GR0023 tgtctcttgcagggctcctgagctgaagtgaagatggtcatactcaaggaagaaccttct

DRB*2_GR0034 tgtctcttgcagggctcctgagctgaagtgaagatggtcatactcaaggaagaaccttct

DRB*2_GR0065 tgtctcttgcagggctcctgagctgaagtgaagatggtcatactcaaggaagaaccttct

DRB*2_GR0140 tgtctcttgcagggctcctgagctgaagtgaagatggtcatactcaaggaagaaccttct

DRB*2_GR0201 tgtctcttgcagggctcctgagctgaagtgaagatggtcatactcaaggaagaaccttct

DRB*2_GR0310 tgtctcttgcagggctcctgagctgaagtgaagatggtcatactcaaggaagaaccttct

DRB*2_GR0616 tgtctcttgcagggctcctgagctgaagtgaagatggtcatactcaaggaagaaccttct

goat_VBN4 tgtctcttgcagggctcctgagctgaagtgaagatggtcatactcaaggaagaaccttct

goat_ALP1.F01 tgtctcttgcagggctcctgagctgaagtgaagatggtcatactcaaggaagaaccttct

goat_ALP1.C02 tgtctcttgcagggctcctgagctgaagtgaagatggtcacactcaaggaagaaccttct

goat_GRS.A04 tgtctcttgcagggctcctgagctgaagtgaagatggtcacactcaaggaagaaccttct

++ 3' UTR ++++++++++++++++++++++++++++++++++++++++++++++++++

DRB*1_GR0150 gtnccagnttcttcncngcanngaaaggtttcctgcttagcgctaactnttccacaatga

DRB*1_GR0701 gtcccagcttcttcacagcatggaaaggtttcctgcttagcgctaactcttccacaatga

DRB*1_GR0721 gtcccagcttcttcacagcatggaaaggtttcctgcttagcgctaactcttccacaatga

DRB*1_GR0732 gtcccagcttcttcacagcatggaaaggtttcctgcttagcgctaactcttccacaatga

DRB*1_VD0030 gtcccagcttcttcacagcatggaaaggtttcctgcttagcgctaactcttccacaatga

DRB*1_VS0112 gtcccagcttcttcacagcatggaaaggtttcctgcttagcgctaactcttccacaatga

DRB*1_VS0139 gtnccagnttcttcacagcatggannngtttcctgcttagcgctaactcttncacaatga

DRB*2_GR0023 gtcccagcttcttcacagtgtggaaaggtctcctgcttagcgctaactcttctacaatga

DRB*2_GR0034 gtcccagcttcttcacagtgtggaaaggtctcctgcttagcgctaactcttctacaatga

DRB*2_GR0065 gtcccagcttcttcacagtgtggaaaggtctcctgcttagcgctaactcttctacaatga

DRB*2_GR0140 gtcccagcttcttcacagtgtggaaaggtctcctgcttagcgctaactcttctacaatga

DRB*2_GR0201 gtcccagcttcttcacagtgtggaaaggtctcctgcttagcgctaactcttctacaatga

DRB*2_GR0310 gtcccagcttcttcacagtgtggaaaggtctcctgcttagcgctaactcttctacaatga

DRB*2_GR0616 gtcccagcttcttcacagtgtggaaaggtctcctgcttagcgctaactcttctacaatga

goat_VBN4 gtcccagcttcttcacagtgtggaaaggtctcctgcttagcgctaactcttctacaatga

goat_ALP1.F01 gtcccagcttcttcacagtgtggaaaggtctcctgcttagcgctaactcttctacaatga

goat_ALP1.C02 gtcccagcttcttcacagcatggaaaggtttcctgcttagcgctgactcttccacaatga

goat_GRS.A04 gtcccngcttcttcacagcatggaaagntttcctgcttagcgntgactcttccacaatga

++ 3' UTR ++++++++++++++++++++++++++++++++++++++++++++++++++

DRB*1_GR0150 agtactttctcaggacctcgtttgctcctggctcagtgaccctgtagaaactgtcctcga

DRB*1_GR0701 agtactttctcaggacctcgtttgctcctggctcagtgaccctgtagaaactgtcctcga

DRB*1_GR0721 agtactttctcaggacctcgtttgctcctggctcagtgaccctgtagaaactgtcctcga

DRB*1_GR0732 agtactttctcaggacctcgtttgctcctggctcagtgaccctgtagaaactgtcctcga

DRB*1_VD0030 agtactttctcaggacctcgtttgctcctggctcagtgaccctgtagaaactgtcctcga

DRB*1_VS0112 agtactttctcaggacctcgtttgctcctggctcagtgaccctgtagaaactgtcctcga

DRB*1_VS0139 agtactttctcaggacctcgtttgctcctggctcagtgaccctgtagaaactgtcctcga

DRB*2_GR0023 agtattttctcaggatctcatttgctcctggctcagtgaccctgcagaaactgtcctcaa

DRB*2_GR0034 agtattttctcaggatctcatttgctcctggctcagtgaccctgcagaaactgtcctcaa

DRB*2_GR0065 agtattttctcaggatctcatttgctcctggctcagtgaccctgcagaaactgtcctcaa

DRB*2_GR0140 agtattttctcaggatctcatttgctcctggctcagtgaccctgcagaaactgtcctcaa

DRB*2_GR0201 agtattttctcaggatctcatttgctcctggctcagtgaccctgcagaaactgtcctcaa

DRB*2_GR0310 agtattttctcaggatctcatttgctcctggctcagtgaccctgcagaaactgtcctcaa

DRB*2_GR0616 agtattttctcaggatctcatttgctcctggctcagtgaccctgcagaaactgtcctcaa

goat_VBN4 agtattttctcaggatctcatttgctcctggctcagtgaccctgcagaaactgtcctcaa

goat_ALP1.F01 agtattttctcaggatctcatttgctcctggctcagtgaccctgcagaaactgtcctcaa

goat_ALP1.C02 agtactttctcaggatctcatttgctcctggctcagtgaccctgtagaaactgtcctcga

goat_GRS.A04 agtactttctcaggatntcatttgctcctggctcagtgaccctgtagaaactgtcctcga

++ 3' UTR ++++++++++++++++++++++++++++++++++++++++++++++++++

DRB*1_GR0150 tggctttctcagtcacctcca-cccccctgccctcagcctttgacctggaagttctcaat

DRB*1_GR0701 tggctttctcagtcacctcca-cccccctgccctcagcctttgacctggaagttctcaat

DRB*1_GR0721 tggctttctcagtcacctcca-cccccctgccctcagcctttgacctggaagttctcaat

DRB*1_GR0732 tggctttctcagtcacctcca-cccccctgccctcagcctttgacctggaagttctcaat

DRB*1_VD0030 tggctttctcagtcacctcca-cccccctgccctcagcctttgacctggaagttctcaat

DRB*1_VS0112 tggctttctcagtcacctcca-cccccctgccctcagcctttgacctggaagttctcaat

DRB*1_VS0139 tggctttctcagtcacctcca-cccccctgccctcagcctttgacctggaagttctcaat

DRB*2_GR0023 tggctttctcagtcaccccca-cccccctaccctcagcctttgacctggaagttctcaat

DRB*2_GR0034 tggctttctcagtcaccccca-cccccctaccctcagcctttgacctggaagttctcaat

DRB*2_GR0065 tggctttctcagtcaccccca-cccccctaccctcagcctttgacctggaagttctcaat

DRB*2_GR0140 tggctttctcagtcaccccca-cccccctaccctcagcctttgacctggaagttctcaat

DRB*2_GR0201 tggctttctcagtcaccccca-cccccctaccctcagcctttgacctggaagttctcaat

DRB*2_GR0310 tggctttctcagtcaccccca-cccccctaccctcagcctttgacctggaagttctcaat

DRB*2_GR0616 tggctttctcagtcaccccca-cccccctaccctcagcctttgacctggaagttctcaat

goat_VBN4 tggctttctcagtcacccccaccccccctaccctcagcctttgacctggaagttctcaat

goat_ALP1.F01 tggctttctcagtcaccccca-cccccctaccctcagcctttgacctggaagttctcaat

goat_ALP1.C02 tggctttctcagtcaccccca-cccccctgccctcagcctttgacctggaagttctcagt

goat_GRS.A04 tggctttctcagtcacnncca-cccccctgccctcagcctttgacctggaagttctcagt

++ 3' UTR ++++++++++++++++++++++++++++++++++++++++++++++++++

DRB*1_GR0150 attgattccagtaccttatgt-tctttcttccttggttccctttgttttcaacttctgtt

DRB*1_GR0701 attgattccagtaccttatgt-tctttcttccttggttccctttgttttcaacttctgtt

DRB*1_GR0721 attgattccagtaccttatgt-tctttcttccttggttccctttgttttcaacttctgtt

DRB*1_GR0732 attgattccagtaccttatgt-tctttcttccttggttccctttgttttcaacttctgtt

DRB*1_VD0030 attgattccagtaccttatgt-tctttcttccttggttccctttgttttcaacttctgtt

DRB*1_VS0112 attgattccagtaccttatgt-tctttcttccttggttccctttgttttcaacttctgtt

DRB*1_VS0139 attgattccagtaccttatgt-tctttcttccttggttccctttgttttcaacttctgtt

DRB*2_GR0023 gttgattccagtaccgtatgt-tctttcttccttggttccctttgttttcaacttctgtt

DRB*2_GR0034 gttgattccagtaccgtatgt-tctttcttccttggttccctttgttttcaacttctgtt

DRB*2_GR0065 gttgattccagtaccgtatgt-tctttcttccttggttccctttgttttcaacttctgtt

DRB*2_GR0140 gttgattccagtaccgtatgt-tctttcttccttggttccctttgttttcaacttctgtt

DRB*2_GR0201 gttgattccagtaccgtatgt-tctttcttccttggttccctttgttttcaacttctgtt

DRB*2_GR0310 gttgattccagtaccgtatgt-tctttcttccttggttccctttgttttcaacttctgtt

DRB*2_GR0616 gttgattccagtaccgtatgt-tctttcttccttggttccctttgttttcaacttctgtt

goat_VBN4 gttgattccagtaccgtatgt-tctttcttccttggttccctttgttttcaacttctgtt

goat_ALP1.F01 gttgattccagtaccgtatgt-tctttcttccttggttccctttgttttcaacttctgtt

goat_ALP1.C02 attgataccagtaccttatgt-tctttcttccttcgttccctttgtttgcaacttctgtt

goat_GRS.A04 attgataccagtaccttatgt-tctttcttccttcattccctttgtttgcaacttctgtt

+++

DRB*1_GR0150 tcc

DRB*1_GR0701 tcc

DRB*1_GR0721 tcc

DRB*1_GR0732 tcc

DRB*1_VD0030 tcc

DRB*1_VS0112 tcc

DRB*1_VS0139 tcc

DRB*2_GR0023 tcc

DRB*2_GR0034 tcc

DRB*2_GR0065 tcc

DRB*2_GR0140 tcc

DRB*2_GR0201 tcc

DRB*2_GR0310 tcc

DRB*2_GR0616 tcc

goat_VBN4 tcc

goat_ALP1.F01 tcc

goat_ALP1.C02 tcc

goat_GRS.A04 tcc
